# The cell type resolved mouse transcriptome in neuron-enriched brain tissues from the hippocampus and cerebellum during prion disease

**DOI:** 10.1038/s41598-018-37715-z

**Published:** 2019-01-31

**Authors:** Anna Majer, Sarah J. Medina, Debra Sorensen, Matthew J. Martin, Kathy L. Frost, Clark Phillipson, Kathy Manguiat, Stephanie A. Booth

**Affiliations:** 1 Zoonotic Diseases and Special Pathogens, National Microbiology Laboratory, Canadian Science Centre for Human and Animal Health, Public Health Agency of Canada, Winnipeg, Manitoba Canada; 2 Viral Diseases, National Microbiology Laboratory, Canadian Science Centre for Human and Animal Health, Public Health Agency of Canada, Winnipeg, Manitoba Canada; 3 Department of Medical Microbiology and Infectious Diseases, College of Medicine, Faculty of Health Sciences, University of Manitoba, Winnipeg, Manitoba Canada

## Abstract

Multiple cell types and complex connection networks are an intrinsic feature of brain tissue. In this study we used expression profiling of specific microscopic regions of heterogeneous tissue sections isolated by laser capture microdissection (LCM) to determine insights into the molecular basis of brain pathology in prion disease. Temporal profiles in two mouse models of prion disease, bovine spongiform encephalopathy (BSE) and a mouse-adapted strain of scrapie (RML) were performed in microdissected regions of the CA1 hippocampus and granular layer of the cerebellum which are both enriched in neuronal cell bodies. We noted that during clinical disease the number of activated microglia and astrocytes that occur in these areas are increased, thereby likely diluting the neuronal gene expression signature. We performed a comparative analysis with gene expression profiles determined from isolated populations of neurons, microglia and astrocytes to identify transcripts that are enriched in each of these cell types. Although the incubation periods of these two models are quite different, over 300 days for BSE and ~160 days for RML scrapie, these regional microdissections revealed broadly similar profiles. Microglial and astrocyte-enriched genes contributed a profound inflammatory profile consisting of inflammatory cytokines, genes related to phagocytosis, proteolysis and genes coding for extracellular matrix proteins. CA1 pyramidal neurons displayed a net upregulation of transcription factors and stress induced genes at pre-clinical stages of disease while all tissues showed profound decrease of overlapping genes related to neuronal function, in particular transcripts related to neuronal communication including glutamate receptors, phosphatase subunits and numerous synapse-related markers. Of note, we found a small number of genes expressed in neurons that were upregulated during clinical disease including, COX6A2, FZD9, RXRG and SOX11, that may be biomarkers of neurodegeneration.

## Introduction

Transmissible spongiform encephalopathies (TSEs), or prion diseases are a group of neurodegenerative diseases that are associated with conversion of the normal form of the prion protein, PrP^C^ (cellular prion protein), to an infectious conformer, PrP^Sc^ (Scrapie prion protein)^1^. Progressive pathology accompanies this refolding including synaptic loss and dysfunction, microgliosis, astrocytosis, vacuolation and eventually, neuronal death. Most of these changes occur progressively over a long pre-clinical incubation period and are irreversible by the time diagnosis occurs. A greater understanding of the molecular changes that underpin this neuropathology would direct the design of therapeutics required to protect and counter the damage to neurons as well as providing some pre-clinical markers that would enable more timely treatment to be initiated.

A number of studies that identify transcriptomic changes in the brains of animals during prion disease using various prion strains and animal models have been published^2–6^. It is clear that the overarching finding in these studies is a progressive increase in gene expression relating to glial activation and proliferation that occurs concomitant with some decreases in expression of genes relating to synaptic function and loss of neurons. However, resolving those specific molecular pathways that lead directly to the degeneration of neurons and the consequential advancement of clinical disease is difficult^7^. A number of approaches can be taken to begin to unravel these molecular changes and determine their temporal role in the biological processes that are at play during neurodegeneration. These approaches can involve both experimental adaptations to determine altered transcriptomes in specific cells affected by disease, such as cell fractionation and tissue microdissection, or bioinformatic approaches to assign changes that occur in specific processes, cells or pathways by comparison with other published datasets. In one of the first such studies, we used laser capture microdissection (LCM) to track the temporal transcriptome in the CA1 region of mouse hippocampus, a region that contains relatively densely packed neuron cell bodies, during infection with RML scrapie^8^. This methodology allowed us to discriminate a considerable number of gene expression alterations that were specific to neurons, as the region dissected remained relatively free of activated glia until extensive neuronal death in the region at the late clinical stage of disease. Temporal transcriptional changes in affected neurons were therefore mapped more accurately than has previously been possible revealing gene signatures reflecting chronic over-activation of neurons, changes to dendritic morphology, and modulation of the unfolded protein response at early stages of disease followed by loss of synaptic and neuronal structural proteins during clinical disease. In addition, we were clearly able to resolve an inflammatory profile during the clinical stage of disease that reflected the infiltration of activated glia into the region following the death and damage of CA1 neurons.

In the current study we extend our previous work to include analysis of the transcriptome within a second brain region enriched with neuronal cell bodies, the granule layer of the cerebellum, and investigated changes within these regions in a second prion infection model, mouse adapted Bovine Spongiform Encephalopathy (BSE). In addition to the precise region-specific temporal information provided by microdissection, we use a bioinformatics protocol to further discriminate those disease-related genes whose expression is particularly enriched in specific cell types; namely, neurons, astrocytes and microglia. Following this approach, we begin to resolve a map of transcriptional changes specific to prion disease that occur in each of the four major cell types in the brain during disease.

## Materials and Methods

### Ethics Statement

All procedures involving live animals were approved by the Canadian Science Centre for Human and Animal Health - Animal Care Committee (CSCHAH-ACC) according to the guidelines set by the Canadian Council on Animal Care. All protocols were designed to minimize animal discomfort. The approval identifications for this study were animal use document (AUD) #’s H04-016, H11-020 and H15-032.

### Animal experiments and brain tissue sampling

RML scrapie infection of CD1 mice was performed as described and mice were sacrificed at five time-points spanning very early to terminal disease [70, 90, 110, 130 and terminal 153–161 days post infection (DPI)]^8^. In a second experiment from which tissues for qRT-PCR validation of microarray data was collected, terminal disease occurred between 169–190 DPI. A total of 4–6 mice per condition per time point were used for these experiments.

Similarly, BSE infection was performed in c57/BL6 mice between 4 and 6 weeks of age using 20 µl of a 10% brain homogenate prepared from a sample of brain tissue from a classical BSE case in a Canadian cow that was generously provided by Dr Stefanie Czub. Following intracranial (i.c.) inoculation 21 of 25 mice were positive for PrP^Sc^ with a prolonged mean survival period (511 ± 73 DPI). Brain tissue from the mouse with the shortest incubation period of 394 DPI was homogenised in PBS and used as inoculum for subsequent passage to produce tissues for transcriptional profiling. In this case, intraperitoneal (i.p.) inoculation of c57Bl/6 mice was performed using 100 µl of 5% brain homogenate and an equal number of control animals were inoculated with 200 µl of 1% brain homogenate collected from healthy age matched c57Bl/6 mouse. Animals were sacrificed in groups of between 4 and 6 infected and mock-infected mice at intervals following inoculation [60, 140, 200, 300 days] and at a terminal time point delineated by the progress of clinical signs including, weight loss of 20% or more, kyphosis and ataxia, which occurred between 304–390 DPI.

Brains were halved and either fixed in formalin for histopathological analysis, or the tissue was frozen in optimal cutting temperature (OCT) medium (Sakura Finetek) and processed for laser capture microdissection of neuron-enriched CA1 and cerebellar granule layer as previously described^8^.

### Microarray gene expression profiling and analysis

Total RNA was isolated from microdissected tissues using RNAqueous–Micro Kit (Life Technologies Inc.) and the RNA concentration and quality was assessed for each sample by Bioanalyzer using the RNA 6000 Pico Kit (Agilent Technologies Inc.). Only samples with RIN value ≥ 6.0 were used for transcriptional profiling (Supplemental Fig. 1). Therefore, for each tissue and time point 4–6 infected and 4–6 control mice were sampled and the isolated total RNA was profiled on Agilent whole mouse genome 4 × 44 K arrays (Agilent Technologies Inc.). Biological replicates constituted pooled RNA isolated from microdissected tissues taken from each individual mouse. Two rounds of amplification were performed using 2 ng of total RNA and the Amino Allyl MessageAmp II aRNA Amplification Kit (Life Technologies Inc.) according to the manufacturer’s instructions. Amplified RNA samples were labeled using Alexa Fluor 647 or 555 (Life Technologies Inc.).

Hybridization and array washes were performed according to the manufacturer’s recommendations and scans were performed using default protocols and settings (Agilent Technologies Inc.). Signal intensities for each probe were quantified and normalized using Agilent Feature Extraction Software versions 9.1 to 10.5.1.1 and only arrays that passed the proprietary QC criteria were further considered in the analysis. This resulted in at least 4 array data sets to be compared for all time points (Gene Expression Omnibus # GSE113697 & GSE34530).

### Data analysis

The total gene signal provided by the Agilent Feature Extraction image analysis software was used for data analysis following filtering to remove features below a minimum intensity threshold. Transcripts that differed in abundance by at least 2-fold during at least one time point throughout infection with a false discovery rate (FDR) of <1% are presented as mean ± standard deviation and analyzed using ANOVA, with p < 0.05 considered as statistically significant. To delineate genes which exhibit enriched expression in microglia, astrocytes, neurons and oligodendrocytes we compared the lists with gene expression data published by Sharma *et al*., 2015 in which deep-sequencing was used to resolve the transcriptomes of CNS cell types^9^. We compared the list of cell-type enriched genes identified in the publication^9^ against genes significantly deregulated at one or more time points in our microarray generated data. Specifically, we assigned a threshold criterion where number of genes/cell-type was over 0.5, indicating at least 50% enrichment within that particular cell type. Those genes were therefore assigned to that cell type and the differentially expressed transcripts across each cell type within the 4 sample types were then temporally evaluated. Venn diagrams were made using the online tool Venny (http://bioinfogp.cnb.csic.es/tools/venny/index.html) to assess the number of deregulated genes for the 4 sample types. Hierarchical cluster plots were produced using GeneMathsXT (www.applied-maths.com) employing the cosine correction and WPGMC (median linkage) measure. Ingenuity Pathway Analysis (Qiagen) was used to identify biological processes that were deregulated throughout diseases for each cell-type.

### qRT-PCR analysis of mRNA gene expression

Total RNA was isolated from laser-captured microdissected samples using the Total RNA Purification Micro Kit (Norgen Biotek Corp.) according to the manufacturer’s instructions. The RNA quality was confirmed using the 2100 Bioanalyzer RNA 6000 Pico Kit (Agilent Technologies Inc.) following manufacturers’ recommendations. Reverse transcription of 2 ng of total RNA was performed using the High Capacity cDNA Reverse Transcriptase Kit (Life Technologies) according to the manufacturers’ protocol. TaqMan RNA Assays were used to validate expression profiles for 6 genes following manufacturers’ recommendations (Life Technologies). GAPDH was used as the endogenous control and fold change was calculated using the 2^−(ΔΔCt)^ method. Standard error and Student’s t-test statistics were calculated to determine significance where p-value < 0.05 was deemed significant.

## Results

### Cell-type resolved transcriptional dynamics during prion disease

**Figure 1:**
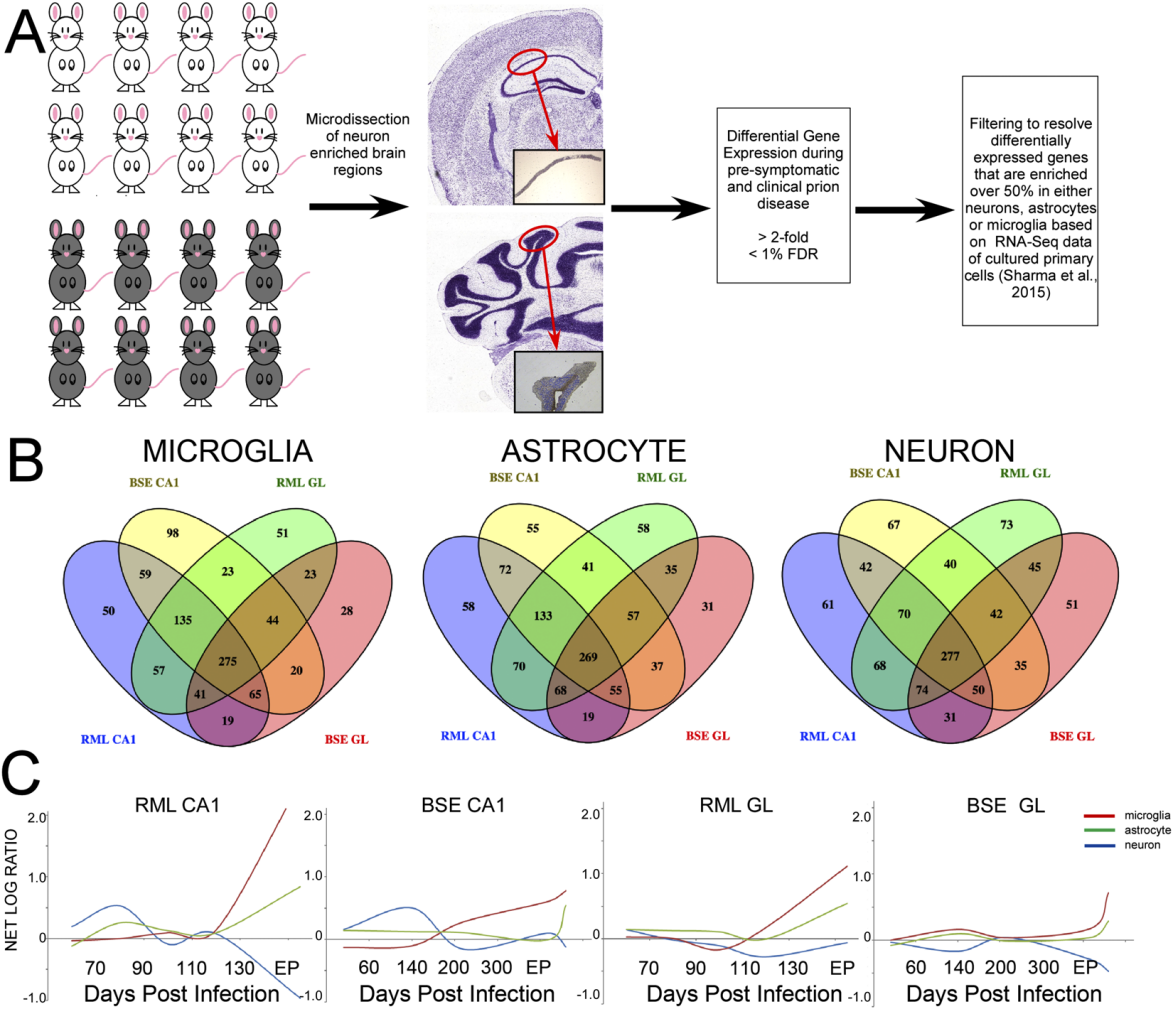
Gene expression dynamics in two regions of mouse brain infected by RML scrapie or BSE prions. (**A**) Schematic illustration of the workflow for the removal of the tissue region of interest and prion strain–resolved temporal transcriptome. (**B**) Venn diagrams illustrating the overlap of differentially expressed genes across excised hippocampal CA1 and cerebellar granule layer regions from mice infected with RML scrapie and BSE prion strains. Diagrams depict comparisons made for microglia-, astrocyte- and neuronal-enriched genes (from left to right). (**C**) Trends in the net temporal expression profiles of microglia- (red), astrocyte- (green), and neuron-enriched genes (blue) across each tissue region and prion strain.

We resolved the transcriptome of two regions of prion-infected mouse brain at various time-points during infection. Mice were intraperitoneally (IP) inoculated with either the RML strain of mouse-adapted prions, or a bovine isolate of BSE passaged once in mice. Controls were mock-infected with brain homogenate from age-matched uninfected mice. Animals were sacrificed at five time-points spanning very early to terminal disease [70, 90, 110, 130 and terminal 153–161 days post infection (DPI) in RML infected mice or 60, 140, 200, 300 and/or terminal 304–390 DPI for BSE]. LCM was used to isolate either pyramidal cells from CA1 hippocampal tissue, or cells from the granule layer of the cerebellum from 8 µm brain sections. Total RNA was extracted and the relative global transcriptome expression levels between infected and control mice (n = 4–6) was determined at each time point. Agilent whole mouse genome microarrays comprising 44,000 probes were used to identify transcripts that differed in abundance by at least 2-fold and a false discovery rate (FDR) of <1% for at least one time point during infection (Fig. 1A**)**. We confirmed the enrichment of genes known to be highly expressed in each region based on the signal intensity of the probe. The relative signal intensities of two such genes, IQGAP2, which is highly expressed in the hippocampal area CA1 and PCP2, a gene that is highly expressed in the cerebellum, are shown in Supplemental Fig. 2.

As the brain is a highly complex tissue containing a vast array of different cell types we consider it imperative to resolve the disease-related transcriptomes of each major cell type. This is especially important during prion-induced neurodegeneration when glial populations exhibit widespread activation and expansion. The marked protein and gene expression alterations that accompany these changes are pervasive within the tissue and mask the subtle regulatory networks within neurons that accompany the slow protracted degeneration that ultimately results in the death of the animal^8^. To examine whether differences in cell fate, i.e. glial expansion and neuronal degeneration at preclinical and clinical phases of disease may affect global gene expression in microdissected areas, we tracked the average signal intensities of neuron, astrocyte, microglia and oligodendrocyte marker genes in each strain and sample type during disease in comparison to age-matched, mock-infected controls. Statistically significant increases in astrocyte and microglial marker genes and reductions of neuronal and some oligodendrocyte markers were evident at later time points (Supplemental Figs 3–6**)**. To provide further clarity, we decided to perform comprehensive comparison of our gene expression data with recently published data sets from Sharma and colleagues (2015) in which deep-sequencing was used to resolve the transcriptomes that are specific to broad central nervous system (CNS) cell types; namely astrocytes, microglia, cortical neurons and oligodendrocytes. We calculated the proportion of each transcript present in each of these cell types to determine cell-type enriched gene profiles. We used the Ingenuity Pathway Analysis (IPA) tool to identify those transcripts that are differentially expressed during prion disease and that are enriched in each cell type. We used a threshold criterion of over 0.5 (50%) enrichment within one particular cell type to assign the differentially expressed transcript to each cell type. We determined very few oligodendrocyte-enriched genes from our list and did not include these in subsequent analysis. The number of genes assigned to each of these specific cell types is shown in Table 1 and the Venn diagrams displayed as Fig. 1B. Enrichment within a particular cell type does not preclude the possibility that disease-related differential expression is occurring in cell types in which their basal expression is less than our 0.5 cut-off value; in fact, very few transcripts are actually truly cell-type specific. However, we believe this broad assignment, along with the technical separation afforded by the microdissection methodology used for sampling, allows these groupings to predict the major trends within each cell type. We found that there was considerable overlap between the differentially represented genes in either prion model and also in each brain region where approximately 40% of genes were in common among all 4 samples, suggesting commonalities between the biological processes that accompany disease within these cell populations, irrespective of the animal model. These gene lists are provided as Supplemental Table 1.

**Table 1:** Differentially expressed genes enriched in the major brain cell-types.

Prion Sample	Microglia-enriched	Astrocyte-enriched	Neuron-enriched	Oligodendrocyte
RML CA1	701	744	673	141
RML GL	649	730	689	148
BSE CA1	720	719	623	149
BSE GL	515	571	605	107
Common to all 4 samples	**275**	**268**	**277**	**38**

Next we determined the net log fold change of all differentially expressed genes enriched in neurons, astrocytes or microglia and plotted this versus the time of sacrifice to identify temporal trends in expression profiles within each cell type (Fig. 1C). Of the two models studied, RML infection is an example of a mouse adapted prion disease with a relatively short incubation period in a *Prnp*^*a*^ genotype mouse line, whereas BSE has a long incubation period in these mice. Overall, net increases and decreases appear more marked in the RML model; as this was only the second passage of BSE in these mice the length of the incubation period had a higher standard deviation. In addition, there is variation between these strains in the regions of the brain in which PrP^res^ accumulation is most dense; scrapie strains are generally denser in all regions of the hippocampus whereas BSE PrP^res^ is less abundant in the hippocampus apart from the CA2 region^10,11^. Similarities between sample groups include a net increase in expression of neuronal genes in the CA1 region of both models during the first half (preclinical) period of disease, and increased expression of glial-enriched genes towards the end of the incubation period. In the RML model a more marked net decrease in neuronal gene expression is apparent in the CA1 region than in BSE infected mice, which appears to show a larger net depletion of neuronal genes in the GL region.

We compared lists of genes enriched in microglia, astrocytes or neurons with a core group of 333 differentially expressed genes central to prion disease identified in a previous comprehensive study. This study determined common differential expressed genes in whole brain tissues of rodents infected with a number of prion strains and was published by Hwang *et al*. in 2009. We found that 321 genes were similarly altered in expression within our microdissected tissues of RML and BSE infected mice analyzed in at least one time point. Of these, 186 were part of the microglia-enriched gene list and 54 were genes expressed more abundantly in astrocytes. Only 4 genes belonged to the neuron-enriched group confirming that the neuronal transcriptome is poorly resolved in RNA extracted from whole brain tissue containing a mixture of cell types.

### Glia-enriched gene expression profiles

**Figure 2:**
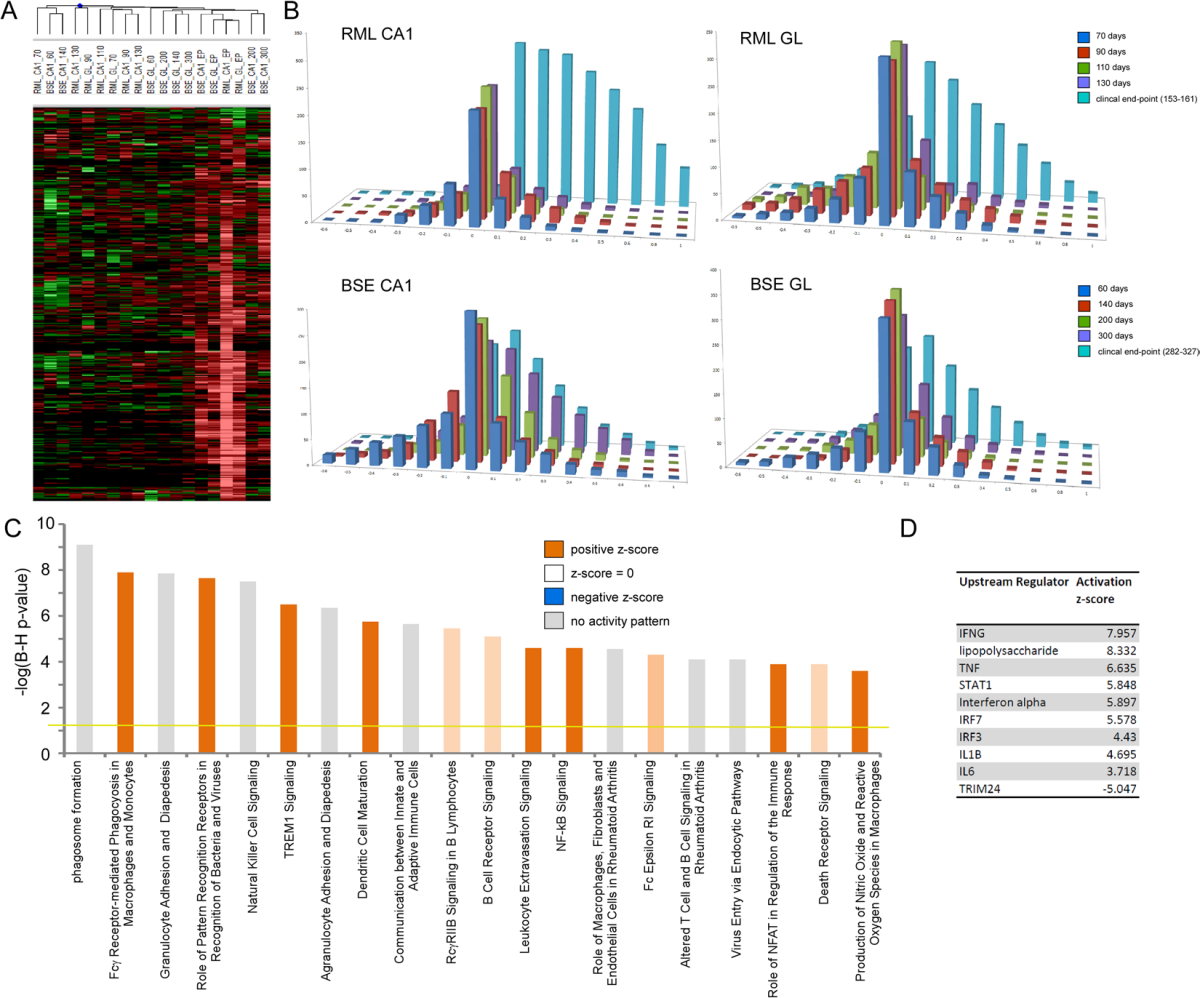
Dynamics and Gene Ontology analysis of genes that are enriched in microglia. (**A**) Heat map of microglia-enriched genes differentially expressed across excised hippocampal CA1 and cerebellar granule layer regions from mice infected with RML scrapie and BSE strains of prions at one or more time-point after unsupervised hierarchical clustering (*n* = 4–6 for each time-point). (**B**) Bar chart shows the number of microglia genes differentially expressed across excised hippocampal CA1 and cerebellar granule layer tissue in infections of mice with RML scrapie and BSE prions. The log ratio for each gene was binned in 0.1 increments along the x-axis, and the number of genes in each bin on the y-axis. (**C**) IPA analysis shows the top 20 canonical pathways that were deregulated by microglia-enriched genes at the clinical end-point of disease. The color of the bars indicates predicted pathway activation based on z-score (orange = activation; blue = inhibition; gray = no prediction can be made; white = z-score close to 0). The horizontal yellow line indicates the p-value threshold. Fisher’s exact test, right-tailed, was used to calculate negative log of p-value. (**D**) IPA analysis shows the top 10 predicted upstream regulators of microglia gene expression at the clinical end-point of disease based on z-score.

A hierarchical clustering showing the kinetics of microglial-enriched gene expression alterations is shown in Fig. 2A and bar charts showing the relative distribution of up and downregulated genes for each prion-disease model and microdissected brain regions are shown in Fig. 2B. Microglial gene markers AIF1, CX3CR1, CD68, TLR2, and TMEM119 are detectable at very low levels in all time points but are significantly increased only after 130 DPI in each model and microdissected region. A number of published studies describe gene expression profiles of microglia in prion disease following isolation from dissociated brain tissue and purification using magnetic beads coated with an antibody to the surface marker CD11b. In total, the majority of the 275 microglia-enriched genes we identified in this study were similarly described in two previous studies^12,13^. We identified a mixture of genes in both animal models at late stage of disease to be associated with both pro-inflammatory and anti-inflammatory responses. For example, we found increased expression of IL1β, TNF-α and CSF1. We also identified upregulation of Signal transducer and activator of transcription (STAT) and Nuclear factor kappa-light-chain-enhancer of activated B cells (NF-kB) responsive genes, pro-inflammatory mediators, and also significant induction of some anti-inflammatory associated genes including CCL2, TGFB1 and the synthetase PTGES that is responsible for the production of anti-inflammatory cytokine Prostaglandin E2. The upstream regulator analysis tool in IPA analyses linkage to differentially expressed genes through coordinated expression to identify potential upstream regulators. These can be transcription factors or any gene or small molecule that has been reported to affect gene expression. In this way we determined the likely upstream regulators of these biological processes (Fig. 2D). As expected, these represent a set of inflammatory regulators including TNF, STAT1 and IL1β.

An IPA analysis was used to determine the most significantly enriched biological processes represented by these microglia-enriched genes at the clinical end-point of disease. These were phagosome formation and Fcγ Receptor-mediated phagocytosis in addition to numerous immune response related signaling pathways. The most significant processes are shown in Fig. 2C. We observed increased expression of TREM2 and TYROBP that form a signaling complex that can enhance the phagocytic activity of microglia during neurodegeneration^14^, and genes such as LCP1, CST7 and CD14 that are strongly associated with phagocytic processes. These likely indicate a generalized response to clearance of protease-resistant misfolded and aggregated proteins. Whilst we know that microglia are activated from very early stages of prion disease, it is not known whether microglia activation is directly caused by contact with accumulating misfolded PrP^sc^ or as a response to synaptic damage. Microglial gene expression profiles in our microdissected tissue samples, most strikingly in the RML model, are only seen after substantial incubation times. This suggests that the resident microglia are activated only when neuronal bodies physically degenerate, which happens only at late stages of disease when clinical signs are apparent. Therefore, it would seem that the microglial response is to accumulating cell debris rather than to a systemic response induced by prion accumulation and signaling from distal regions.

**Figure 3:**
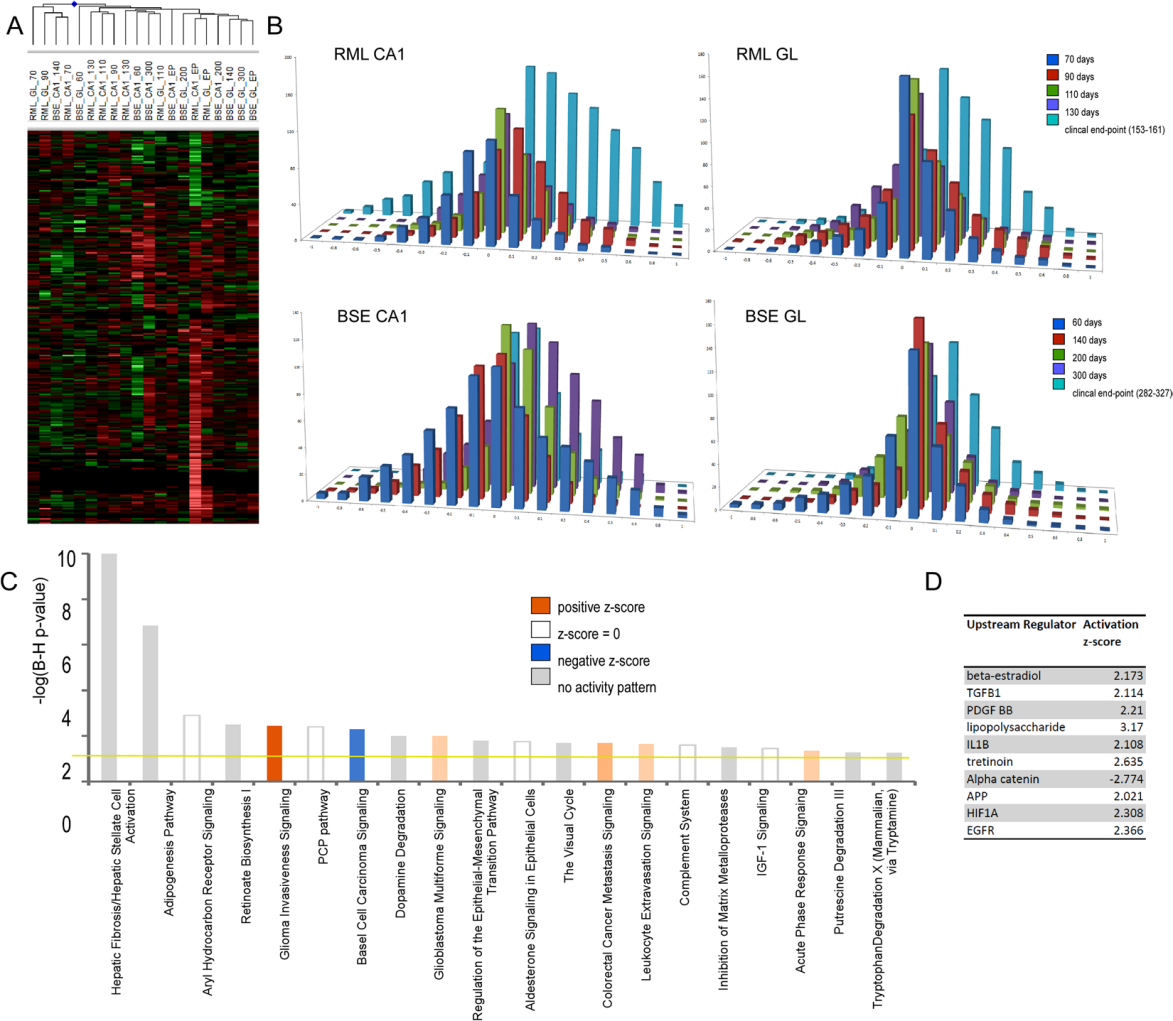
Dynamics and Gene Ontology analysis of genes that are enriched in astrocytes. (**A**) Heat map of astrocyte genes differentially expressed across excised hippocampal CA1 and cerebellar granule layer tissue in infections of mice with RML scrapie and BSE prions at one or more time-point after unsupervised hierarchical clustering (*n* = 4–6 for each time-point). (**B**) Bar chart shows the number of astrocyte genes differentially expressed across excised hippocampal CA1 and cerebellar granule layer tissue in infections of mice with RML scrapie and BSE prions. The log ratio for each gene was binned in 0.1 increments along the x axis, and the number of genes in each bin on the y-axis. (**C**) IPA analysis shows the top 20 canonical pathways that were deregulated by astrocyte enriched genes at the clinical end-point of disease. The color of the bars indicates predicted pathway activation based on z-score (orange = activation; blue = inhibition; gray = no prediction can be made; white = z-score close to 0). The horizontal yellow line indicates the p-value threshold. Fisher’s exact test, right-tailed, was used to calculate negative log of p-value. (**D**) IPA analysis shows the top 10 predicted upstream regulators of astrocyte gene expression at the clinical end-point of disease based on z-score.

Figure 3A shows the corresponding analyses of astrocyte-enriched gene expression. Similar to the microglia profile, the majority of the astrocyte-enriched genes show increased expression indicative of reactive astrocytosis, although some downregulated genes are apparent. Whilst the astrocyte-enriched profiles are similar in the microdissected cerebellar granule region of both the RML and BSE models, the profiles in the CA1 models differ significantly from that seen in microglia (Fig. 3B). Altered expression in astrocyte-enriched genes appears at earlier time-points in both models with both downregulated genes apparent by 60 days in BSE and 70 days in the RML model. A number of genes on this list are also induced by 60 days in the BSE model, however, increased expression is only seen at 90 days in the RML model. Unlike microglia, astrocytes do not proliferate substantially in neurodegenerative disease; an increase between 0–3% has been reported in mouse models of Alzheimer’s disease^15,16^. Nevertheless, astroglial processes may extend into the CA1 region that was microdissected following their activation and astrocyte-specific genes can therefore be detected during early disease. Genes induced include well described markers of reactive astrocytes such as GFAP, CD44 and LCN2, however, these were not induced until the clinical stage of disease in the RML model. Marker genes of mature astrocytes such as VIM and AQP4 were present throughout the incubation period suggesting that resident astrocytes are present in the microdissected tissue but become active only once neuronal cell bodies degenerate.

Astrocyte-enriched genes that were induced fell into a number of groups identified by IPA biological enrichment analysis summarised in Fig. 3C, the most significant being hepatic Fibrosis/Hepatic Stellate Cell Activation and the Adipogenesis Pathway. Many of the proteins were involved in extracellular matrix modification and adhesion including collagen (COL3A1, COL4A1, COL4A2, COL4A6, COL6A1, and COL8A2) and numerous others such as the small leucine-rich proteoglycans Lumican (LUM) and Decorin (DCN). Also induced are genes involved in adhesion (CAV2, IGFBP7, ITGB4, NEDD9, PDLIM7, SYNPO2, PDLIM1, MMP14, FLNB, FBLN7, AHNAK, VCAM1, GLYCAM1) development (SOX9, WNT5A, PDLIM7, STK36, PAX6, PRRX2, FZD2, FZD6, CNTF, NXN, FOXC2, WNT9A and FGF1) and the stress response (HSPB6, HSPA2, HSPB8, HSPB2, HSPB1, DNAJC3). The IPA tool was used to identify upstream regulators of expression of the astrocyte-enriched genes where the most likely regulators were determined to be TGFB1 and inflammatory mediators as well as PDGF-BB, APP and HIF1A (Fig. 3D).

### Neuronal gene expression mirrors synaptic plasticity and excitability changes in preclinical prion disease

**Figure 4:**
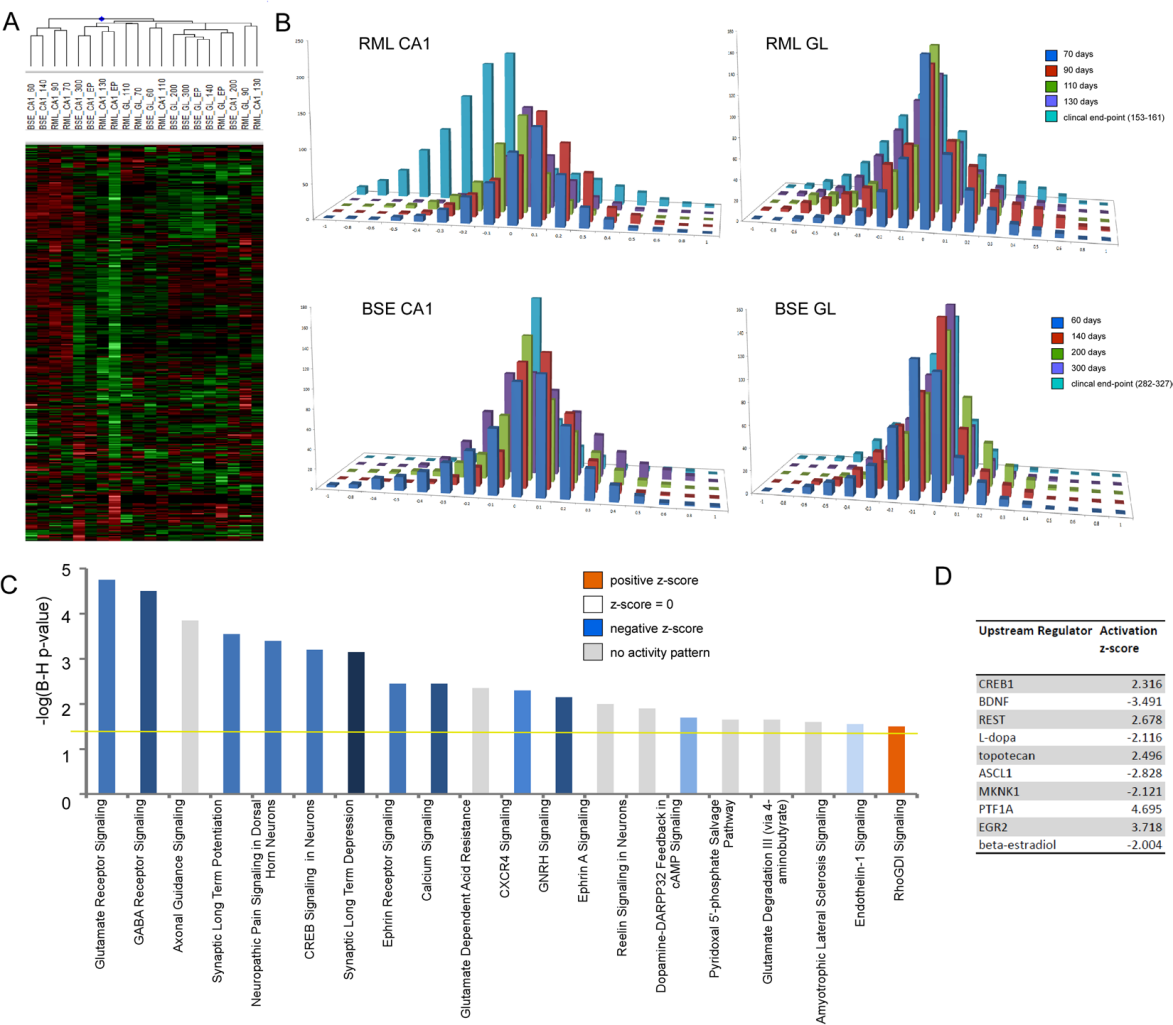
Dynamics and Gene Ontology analysis of genes that are enriched in neurons. (**A**) Heat map of neuron genes differentially expressed across excised hippocampal CA1 and cerebellar granule layer tissue in infections of mice with RML scrapie and BSE prions at one or more time-point after unsupervised hierarchical clustering (*n* = 4–6 for each time-point). (**B**) Bar chart to show the number of neuron genes differentially expressed across excised hippocampal CA1 and cerebellar granule layer tissue in infections of mice with RML scrapie and BSE prions. The log ratio for each gene was binned in 0.1 increments along the x axis, and the number of genes in each bin on the y-axis. (**C**) IPA analysis to show the top 20 canonical pathways that were deregulated by neuron enriched genes at the clinical end-point of disease. The color of the bars indicates predicted pathway activation based on z-score (orange = activation; blue = inhibition; gray = no prediction can be made; white = z-score close to 0). The horizontal yellow line indicates the p-value threshold. Fisher’s exact test, right-tailed, was used to calculate negative log of p-value. (**D**) IPA analysis to show the top 10 predicted upstream regulators of neuron gene expression at the clinical end-point of disease based on z-score.

By selectively removing regions of the brain that are rich in neurons, rather than using whole brain tissue for profiling, we were able to significantly enrich our sample for genes expressed in neurons in comparison to other studies using whole tissue as the starting material for RNA isolation. The hierarchical cluster and bar charts shown in Fig. 4A,B confirm the temporal profile of expression seen in Fig. 1C in which the neuronal signature of the CA1 region exhibits a net increase in the gene expression profile of selected genes prior to significantly decreased expression at the end of the disease process. No net increase is apparent in granule layer neurons however these groups of genes are less abundant at late stages of disease although to a lesser extent, which may be indicative of a lower amount of neuronal degeneration in this long incubation model. An IPA biological enrichment analysis summarised in Fig. 4C indicates that the neuron-enriched genes identified in our prion disease models are primarily localized within neuronal projections and involved in synaptic processes such as glutamate receptor signaling and other signaling pathways involved in neuron function. These integrated functions include behavior, cytoskeleton organization, synaptic transmission, neurite growth and differentiation. Table 2 summarizes some of these altered genes that make up the top 5 ranked molecular networks generated by IPA. We used the  IPA tool to identify upstream regulators of expression of the neuron-enriched genes, which predicted CREB1, REST, BDNF and EGR2 to be positive regulators of neuronal gene expression, with negative regulation suggested by BDNF (Fig. 4D).

**Table 2:** The top 5 ranked molecular networks identified by Ingenuity pathway analysis summarize altered neuronal-enriched genes. Differentially expressed focus genes are shown in bold type.

Molecules	Score	Top Diseases and Functions
Akt, Ampareceptor, **APBA1**, **ARHGAP26**, **CASKIN1**, **CHD5**, **CLSTN2**, **DLG2**, **DLGAP1**, **KALRN**, **LANCL2**, **LIN7A**, **LRP8**, **MYO1B**, NCadherin, **NBEA**, **NLGN1**, **NREP**, **NRXN1**, **PIP4K2B**, **PLXNA2**, **PROK2**, **PROKR2**, **PTBP2**, Secretasegamma, **SEMA3A**, **SLC17A6SNAP25**, **SOX11**, **ST8SIA4**, **STAU2**, **STRBP**, Syntaxin, **SYT1**, **TSPYL5**	56	Cell-To-Cell Signaling and Interaction, Nervous System Development and Function, Neurological Disease
**ACVR2A**, alcoholgroupacceptorphosphotransferase, **ALDH1A3**, **AMPH**, **Ank2**, **CPNE4**, **DLEU7**, **DMXL2**, GABA-A receptor, **GABRA1**, **GABRA2**, **GABRB2**, **GABRG2**, **GAP43**, **GDA**, **HOMER1**, ITPR, **KIFAP3**, **MAP2K6**, MAP3K, **MAP3K9**, **MAP3K10**, Mek, MLK, N-type Calcium Channel, NFkB (complex), Nos, **Nos1ap**, **PAK5**, Pkg, **PRKCE**, **RAB3C**, **TANC2**, **TMOD2**, tubulin (family)	40	Neurological Disease, Organismal Injury and Abnormalities, Connective Tissue Disorders
cacn, **CACNA1H**, **CACNA2D1**, **CACNA2D2**, **CALB2**, Calcineurin A, Calcineurinprotein(s), calpain, **CAMK4**, CaMKII, Creb, ERK, Hdac, **HDAC9**, **KCNC1**, **MAP2**, **MARK1**, MEF2, **MEF2C**, **Nefm**, NFAT (complex), Nfat (family), **NTS**, **PDE10A**, **PGM2L1**, Pkacatalyticsubunit, **PPM1E**, **PPP3CB**, **RUNX1T1**, **RYR2**, **SLC8A1**, **TAC1**, thymidine kinase, **TRO**, **WIF1**	36	Cardiac Enlargement, Cardiovascular Disease, Cardiovascular System Development and Function
Cadherin, **CDH8**, **CDH10**, **CELF2**, **CTNND2**, **DACT1**, **EEF1A2**, EphReceptor, EPHA, **EPHA3**, **EPHA4**, **EPHA5**, **EPHA7**, Fgf, **FGF12**, **FGF19**, Fgfr, **GPC5**, GTPase, Hedgehog, **HPCAL4**, Integrin, JAK1/2, Jnk, **KIT**, **NPAS4**, PLCgamma, Proinsulin, **RABGAP1L**, Rap1, **RSPO2**, Sfk, **SH3GL2**, **SLC17A7**, **TBC1D30**	33	Cancer, Gastrointestinal Disease, Hepatic System Disease
ADCY, **ADCY1**, ADRA1, **Akap9**, Betaadaptin, **CADPS**, Camk, Collagen Alpha1, **CPLX2**, **DOK5**, **ERC2**, ERK1/2, **FGF13**, Gi-coupledreceptor, glutamatereceptor, **GNAL**, **GNAZ**, GRI, GRIA, **GRIA1**, **GRIA2**, **GRIK1**, **GRIP1**, LtypeCalciumChannel, mGluR, **MMD**, **NETO1**, **PCLO**, **PDE2A**, Plcbeta, PlexinA, Rab5, **RAPGEF4**, **RGS4**, Snare	29	Cell-To-Cell Signaling and Interaction, Nervous System Development and Function, Behavior

As we showed in a previous study^8^, many of these genes including GRIA1, GRIA2 have increased expression in CA1 hippocampal neurons during preclincal disease, prior to a generalized profound decrease in expression correlating with degeneration of the neuron cell bodies in the CA1 region and clinical signs in the mice.

### Increased expression of a small subset of neuronal enriched genes

**Figure 5:**
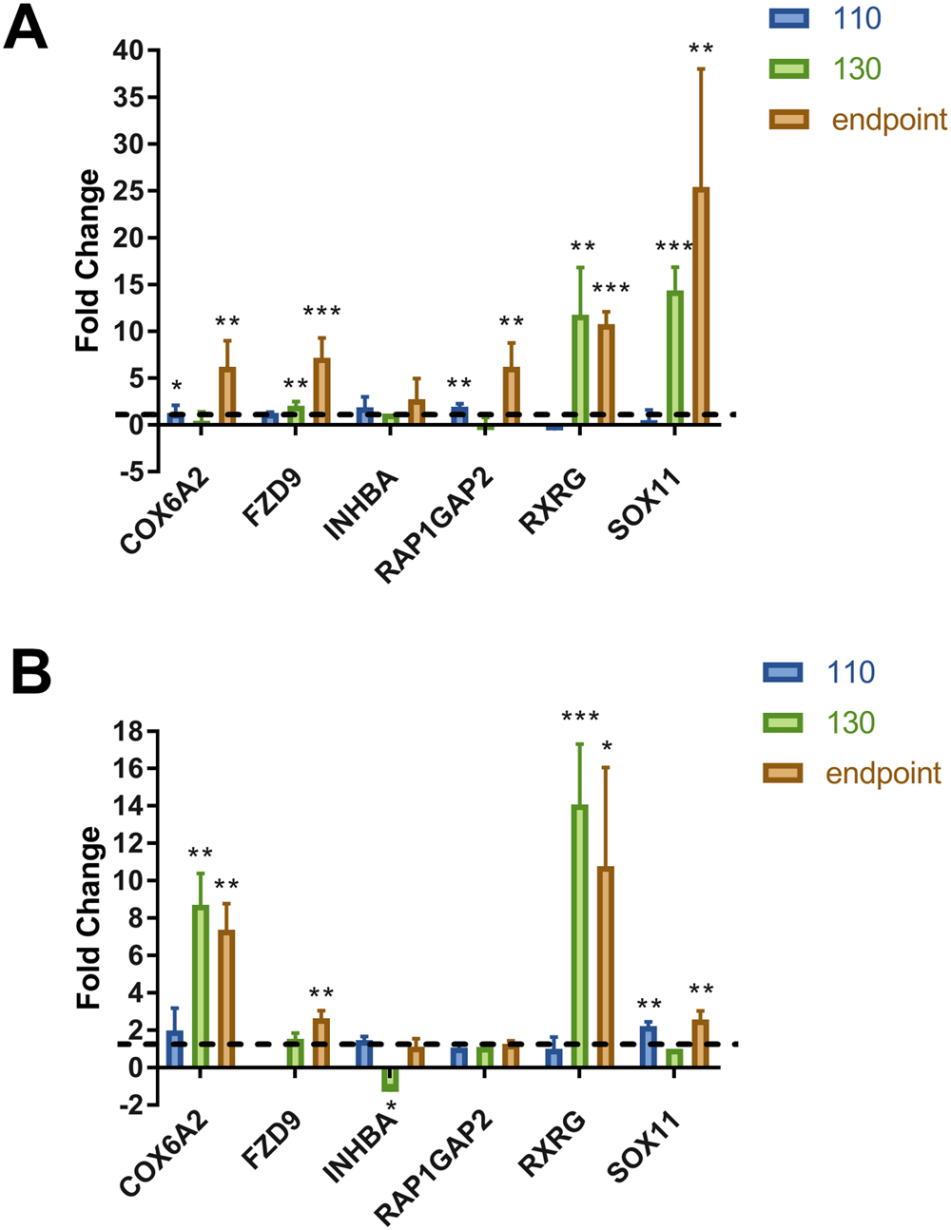
Validation of neuronal-specific genes that were induced during late stages of RML-scrapie infected mice. Fold change of 6 genes were calculated between RML infected and mock-infected (**A**) CA1 hippocampal and (**B**) cerebellar granular layer microdissected regions. GAPDH served as the normalization control for all validations. Data is represented as mean ± SEM (n = 4). A one-tailed unpaired t-test was used to calculate significance where *p < 0.05; **p < 0.01; ***p < 0.001.

At least 95% of the genes whose expression was annotated as either significantly enriched in, or specific to neurons, were progressively less abundant during late pre-clinical and clinical stages of disease. We were particularly interested to note that a small number of neuron enriched-genes were induced concomitant with damage and death of the cells. Induction of these genes could indicate that they play an active role in the damage and degeneration of neurons and therefore could serve as useful biomarkers to identify the onset and progression of disease-related pathologies as well as potentially be targets for therapeutics. We noted 21 Agilent probes (corresponding to 19 genes) within the neuron-enriched list of genes indicating increased expression concomitant with the onset of clinical signs in the mice by the 130 days post-inoculation time-point. These genes are listed in Table 3 along with any reported functions in neurons. We noted that a number of genes within this group have been reported to be involved in adult neurogenesis and neuronal differentiation. We cross referenced these genes with the Allen brain atlas (http://www.brain-map.org/) and BrainStars (B*) (http://brainstars.org/) *in situ* hybridization maps to validate expression of these genes within both the mouse hippocampus CA1 region and the granule layer of the cerebellum, and chose 5 of these genes to validate using qRT-PCR; COX6A, FZD9, RAP1GAP2, RXRG and SOX11. INHBA was also chosen as microarray analysis showed the expression of this gene to be unchanged at the late stages of disease. All of the genes were detected by qRT-PCR in RML infected CA1 and cerebellar granule layer microdissected neurons from tissues infected in a subsequent experiment in which mice reached end-point between 160 and 190 days post-infection. INHBA did not show altered expression relative to the control mice in either tissue at any time-point. However, COX6, FZD9, RXRG and SOX11 all exhibited increased expression in microdissected neuron rich tissues relative to similarly dissected regions from control mice at end-point. Data from CA1 neuron-rich tissue is shown in Fig. 5A and granule layer neuron-rich tissue in Fig. 5B.

**Table 3:** Neuron-enriched genes that showed increased levels during late stages of disease.

Gene Symbol	Gene Name	Biological function (representative GO terms)
RAB6B	RAB6B, member RAS oncogene family	small GTPase mediated signal transduction; protein transport; vesicle-mediated transport
RAP1GAP2	RAP1 GTPase activating protein 2	negative regulation of neuron projection development; regulation of small GTPase mediated signal transduction
RBFOX2	RNA binding protein, fox-1 homolog 2	RNA splicing; radial glia guided migration of Purkinje cell; dendrite morphogenesis
STAC3	SH3 and cysteine rich domain 3	neuromuscular synaptic transmission; intracellular signal transduction
SOX11	SRY (sex determining region Y)-box 11	negative regulation of transcription; nervous system development; positive regulation of cell proliferation; glial cell development
CTNND2	catenin (cadherin associated protein), delta 2	regulation of transcription, regulation of synaptic plasticity; synapse organization; dendritic spine morphogenesis,
CBLN2	cerebellin 2 precursor protein	positive regulation of synapse assembly
COX6A2	cytochrome c oxidase subunit VIa polypeptide 2	mitochondrial electron transport, cytochrome c to oxygen
CISH	cytokine inducible SH2-containing protein	negative regulation of protein kinase activity; protein kinase C-activating G-protein coupled receptor signaling pathway; protein ubiquitination; cytokine-mediated signaling pathway; regulation of growth
FOXO6	forkhead box O6	regulation of transcription; memory; positive regulation of dendritic spine development
FZD9	frizzled class receptor 9	cell surface receptor signaling pathway; learning or memory, Wnt signaling pathway; positive regulation of neural precursor cell proliferation
KIF21B	kinesin family member 21B	microtubule-based movement
RXRG	retinoid X receptor gamma	regulation of transcription; regulation of myelination; retinoic acid receptor signaling pathway
SLC7A3	solute carrier family 7 (cationic amino acid transporter, y + system), member 3	amino acid transmembrane transport
TENM3	teneurin transmembrane protein 3	cell morphogenesis; cell adhesion; positive regulation of neuron projection development; cell differentiation,
SLA	Src like adaptor	cell differentiation; innate immune response;
DNAJB12	DNAJ heat shock protein family (Hsp40) member B12	NRF2-mediated Oxidative Stress Response; Protein Ubiquitination Pathway
HSPB3	Heat-shock protein family B (small) member 3	response to unfolded protein
FAM110D	Family with sequence similarity 110 member D

## Discussion

In this study we determined the temporal transcriptome in the CA1 region of the hippocampus and the granule layer of the cerebellum in two mouse models of prion disease, RML and BSE. Microdissection and bioinformatics allowed us to identify temporal changes in the expression of genes whose expression is enriched in particular cell-types; broadly neurons, astrocytes or microglia. We identified correlative expression profiles in each tissue type and prion strain and determined a subset of genes that are commonly dysregulated in each cell-type.

An activated microglial signature was not apparent until the end stage of disease in the RML model, although some genes were activated earlier in the long incubation period BSE model, most were not apparent until after 140 days incubation. This either reflects the relative stage of activation of resident microglia in the microdissected region or a later influx of activated microglia from surrounding brain tissue. However, the inflammatory profile was not as marked in the BSE model as it was in the RML model perhaps indicating disease related change between these strains in the *Prnp*^*a*^ genotype mouse line. The BSE inoculum used was, however, only the second passage from a BSE infected cow therefore differences in disease process may be due to incomplete strain adaptation in the mouse model. The disease associated transcriptome of microglia was very similar to those published in a number of comprehensive manuscripts on cells isolated from rodent models. Genes identified in our study were indicative of a largely inflammatory profile highly similar to that described in the literature^5,12,17^. We found that the most significantly induced biological processes were phagocytosis and proteolysis, indicating a microglial role in the removal of cellular debris. We also noted induction of some neuroprotective factors such as IGF1, TREM2 and TYROBP. IGF1 has been linked to neuronal survival in cell culture models of prion disease and is a factor that has been shown to be neuroprotective in other models of neurodegenerative disease, such as ALS^18,19^. TREM2 is a transmembrane protein that interacts directly with TYROB, a downstream adapter molecule, which are both also upregulated in a mouse model of Alzheimer’s disease. These proteins are believed to be neuroprotective by triggering an increase in phagocytosis and concomitant suppression of proinflammatory cytokine production^20,21^. Mutations within TRIM2 have been linked to susceptibility to Alzheimer’s disease, however, one variant R47H was not found to be linked to human prion disease^22,23^. In mouse scrapie TREM2 overexpression, although playing a role in microglial activation, was found not to contribute to prion pathogenesis^24^. Microglia can adopt numerous functionally different phenotypes and activation may occur in two or more stages and depending on the exact nature of the stimulus. Therefore, a single cell approach to analysis would be required to shed further light on these processes.

The list of astrocyte-enriched genes revealed the extensive induction of genes that make up the extracellular matrix that occurs during reactive astrocytosis such as versican, aggrecans and chondroitin sulfate proteoglycan^25^, as well as genes involved in various signaling pathways associated with glioma maturation, such as SOX9^26,27^. In adult brain, SOX9 expression is confined to astrocytes^28^, and appears to function in the regulation of astrogliosis in response to brain injury^29,30^. The transcription regulator CTNNB1, growth factor TGFB and the cytokine TNF are predicted as major upstream regulators of astrocyte-enriched genes. CTNNB1 is a transcription factor that is located on the surface of many different types of cells and has been shown to be an important regulator of the Wnt pathway and migratory phenotype in epithelial cells following injury^31^. Activation at the cell membrane results in its nuclear translocation and the transcription of several genes favoring proliferation such as Frizzled and LDL receptor related proteins and other Wnt family regulators^32^. In fact the top 2 generated networks of inter-related astrocyte-enriched genes predicted by IPA involve the activation of these genes. Reactive astrocytes secrete TGF-β, and this has also been linked to protein aggregation and neurodegenerative pathways in ALS and AD^33,34^. Notably, in this study TGF-β1 prevented hippocampal dendritic spine loss and memory impairment in mice that received an intracerebroventricular infusion of amyloid-β oligomers suggesting that astrocyte-derived TGF-β1 is part of a neuroprotective mechanism that protects synapses.

In a previous study we showed that neuronal bodies within the CA1 hippocampal region do not show significant degeneration during preclinical disease and activated glia do not significantly infiltrate the tightly packed neuronal layers until late in the disease process. This is despite the fact that activation of microglia occurs throughout the brain shortly following prion neuroinvasion. In this study, similar findings were apparent in the cerebellar granule neuron layer and in the BSE infection model. Some differences are evident, however, and as previously described the two models used exhibit different incubation periods and variations in the pathology observed. In addition two different mouse strains, the outbred line CD1 and inbred line c57/Bl6 were used and there are known to be neuroanatomical differences between these strains. For example 3D MRI has shown increased brain volume in C57/BL6 mice versus CD1 mice and these strains have also been shown to respond differently to some neuronal challenges^35,36^. It is possible that such strain differences could be reflected in variations in transcription in the different brain regions. However, the transcriptional profile between the microdissected CA1 region from C57BL and CD1 wild-type mice are very similar, <1% of genes are altered between strains more than 1.5-fold and FDR <1% (data not shown). Therefore we concluded that majority of the transcriptional changes in disease that were shared in the two models are more strongly associated with infection than reflective mouse strain. Microdissection of these tissue regions appears to be an excellent method to determine altered gene expression specific to neurons during preclinical disease.

**Figure 6:**
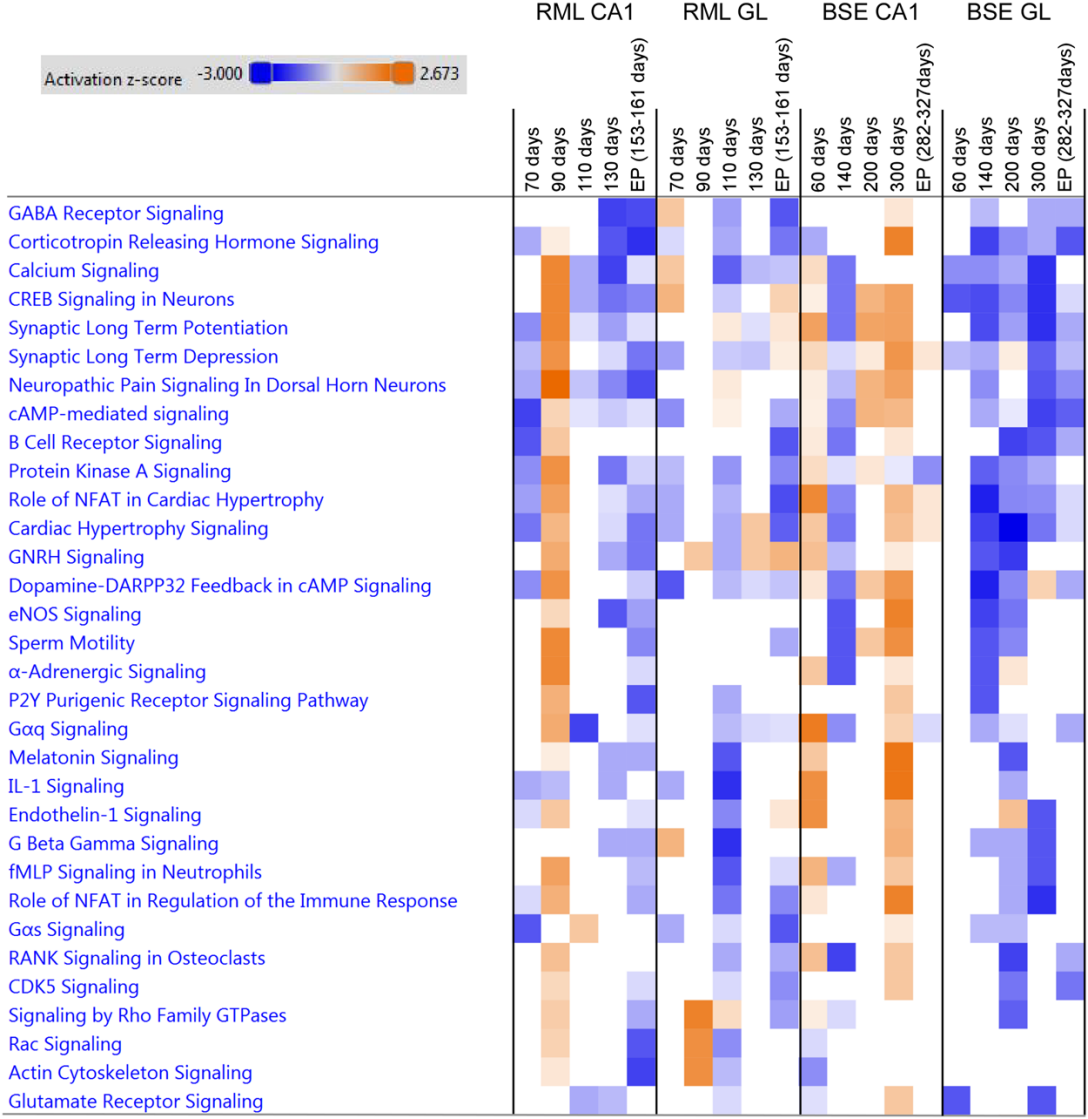
Comparison analysis of canonical signaling pathways temporally activated in neurons during prion infection. Heat map showing top activated canonical pathways generated using genes with expression enriched in neurons during prion infections. Upregulated pathways are shaded orange, down-regulated pathways are shaded blue. The intensity indicates the degree that each gene was up-regulated or down-regulated as determined by the IPA determined z-score.

In this study we found similar gene expression changes occurred in the CA1 neurons in a BSE mouse model during preclinical disease when compared to the pre-clinical gene expression alterations we previously reported in CA1 hippocampal neurons of RML infected mice in a previous study^8^. Here we also identified a subset of genes with altered expression that was common to neurons within the cerebellar granule layer in both mouse models. We found that many of the significantly induced biological processes were involved in dendritic spine remodeling and cell signaling receptor mediated pathways. Early transcriptional changes in CA1 neurons suggest NMDAR-dependent excitability of hippocampal neurons and dendrite and synaptic remodeling, processes that have also been implicated in AD^37,38^. To illustrate these temporally dynamic changes in gene expression we generated a heat map to compare the temporally activated canonical pathways during prion infection from each sample type, which is shown in Fig. 6.

Of particular interest was the finding of a small subset of neuron-enriched genes for which the abundance was elevated late in disease, corresponding to a time when neuron cell bodies are beginning to be lost in these regions. It is possible that the induction of these genes could play an active role in the damage and degeneration of the neurons. The fact they are induced whereas the majority of neuron-enriched genes identified as dysregulated exhibit marked decrease in expression, suggests that they could be useful as biomarkers to identify the onset and progression of disease-related pathologies. In total, 19 neuronal-specific genes were increased in expression according to the microarray data. Gene ontology analysis revealed that 6 (CBLN2, CTNND2, FOXO6, FZD9, RBFOX2 and SOX11) play a role in neuronal development.

We chose 5 genes for further validation by qRT-PCR; COX6A, FZD9, RAP1GAP2, RXRG and SOX11. All exhibited increased expression at the terminal stage of disease in CA1 neurons, and four of the 5 (COX6A, FZD9, RXRG and SOX11) in the cerebellar granule layer in RML infected mice. Both SOX11 and FZD are involved in the development of neurons. SOX11 is a member of the SoxC family of transcription factors that plays a role in neuronal differentiation in several species and a number of different regions of the nervous system including the adult mouse hippocampus^39^, the mouse cortex^40,41^, and as an enhancer of axonal growth and survival in mouse sympathetic neurons^42,43^. The constitutive knockout of Sox11 is lethal^44^, whereas conditional knockout results in mice with fewer neurons in the brain^41^. Interestingly, overexpression of Sox11 has recently been shown to promote neurogenesis from adult hippocampal neurons and may therefore be important for neuronal regeneration after damage or disease^39^. Indeed, over expression of SOX11 in the injured spinal cords of mice was recently shown to result in a marked improvement in locomotor recovery with evidence pointing to increased differentiation and migration of neuronal stem cells to the injury site^45^. In another study, mouse SOX11 was found to control neuronal morphology and its knock-out or overexpression in adult cortical neurons resulted in changes in dendritic complexity^46^. This analysis revealed that long-term elevation of SOX11 resulted in decreased dendritic branching and complexity compared to control neurons, although switching on and off of expression at different stages of neuronal development in culture resulted in opposite effects. SOX11 may potentially have a role in prion induced neurodegeneration by modulating dendritic branching in response to damage during the disease process. FZD9 is one of a family of 10 unconventional G protein-coupled Wnt receptors that mediate signalling via a number of different ligands^47^. FZD9 function has not been well described in the literature to date but has recently been identified as a mediator of Wnt-5a signaling, mediating spine formation in hippocampal neurons and as having a key role in the formation of neuronal connectivity^48,49^. It is localized in the growth cones of adult neurons that are regenerating neurites in response to damage^50^.

RXRG is expressed highly in the CA1 region of the hippocampus and encodes a member of the retinoid X receptor family of nuclear receptors. Interestingly, an agonist of RXRG, bexarotene, has been the focus of numerous recent studies in which it has been used to treat disease in mouse models of AD, Parkinson’s, ALS, multiple sclerosis and stroke^51–54^. Results have showed improved memory, cognition as well as increased neuronal survival and improved pathology, such as plaque removal in AD models^55–57^.

In summary, applying a comparison approach to remove glial-enriched gene expression profiles from highly-complex sample provides an option to determine biological processes induced by disease that are unique to neurons. Although this obviates general effector genes that are important in, and therefore common to numerous cell types it enables the identification of genes that are specific to neurons. In this way we have identified some candidate genes that could act as biomarkers to track the progression of the neurodegenerative process itself. These profiles also provide further insight into the transcriptome that underlies prion replication associated neurodegeneration. Further advances in technology that enable the deconstruction of transcriptome expression at the level of single cells will be key to understand cellular phenotypic responses to prion disease. This type of approach will enable further advances in understanding the neurodegenerative process, such as the determination of the molecular basis of the phenomenon of selective vulnerability, whereby specific brain regions and neuronal cell types are selectively targeted by different prion strains.

## Supplementary information

Supplementary Data
