# Supplementary material for: The cell type resolved mouse transcriptome in neuron-enriched brain tissues from the hippocampus and cerebellum during prion disease
Source: Sci Rep. 2019 Jan 31;9:1099. doi: 10.1038/s41598-018-37715-z (PMC6355796; doi:10.1038/s41598-018-37715-z)
Supplement: Supplementary file 1 — Supplementary Data [file 41598_2018_37715_MOESM1_ESM.pdf]

The cell type resolved mouse transcriptome in neuron-enriched brain tissues from the hippocampus and cerebellum during prion disease

Anna Majer[1,2], Sarah J. Medina[1], Debra Sorensen[1], Matthew Martin[1,3], Kathy L. Frost[1], Clark Phillipson[1], Kathy Manguiat[1], Stephanie A Booth[1,3 *]

1. Zoonotic Diseases and Special Pathogens, National Microbiology Laboratory, Canadian Science Centre for Human and Animal Health, Public Health Agency of Canada, Winnipeg, Manitoba, Canada
2. Viral Diseases, National Microbiology Laboratory, Canadian Science Centre for Human and Animal Health, Public Health Agency of Canada, Winnipeg, Manitoba, Canada
3. Department of Medical Microbiology and Infectious Diseases, College of Medicine, Faculty of Health Sciences, University of Manitoba, Winnipeg, Manitoba, Canada

*Corresponding author
Dr. Stephanie Booth, Zoonotic Diseases and Special Pathogens, National Microbiology Laboratory, Canadian Science Centre for Human and Animal Health, Public Health Agency of Canada, Winnipeg, Manitoba, Canada; E-mail: Stephanie.Booth@canada.ca (SAB);
Phone: +1 204 789 6031.

Supplemental Figure 1. Bioanalyzer readings of representative samples microdissected from prion infected brain tissue.

RIN values indicating quality of the sample are depicted for the granular layer from (A) BSE and (B) RML infected mice. Samples collected from the CA1 hippocampal region for (C) BSE and (D) RML infected mice. The days post inoculation (DPI) time point is indicated for each samples in parentheses where EP represents the end-point of infection or terminal disease.

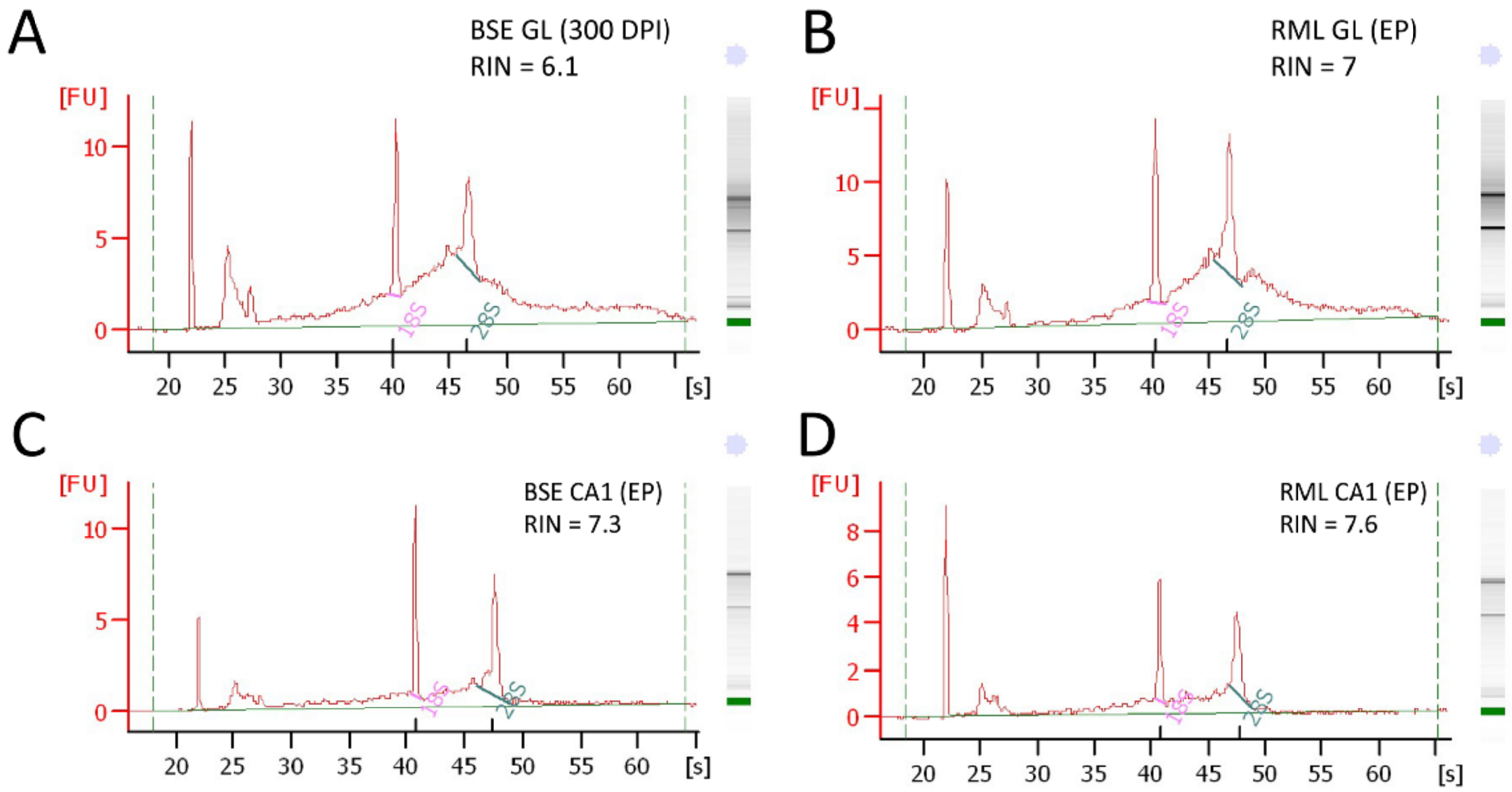

Supplemental Figure 2. Relative signal intensities of IQGAP2 and PCP2 genes that are highly expressed in the hippocampal and cerebellum regions respectively.

We chose 2 genes that show highly enriched expression in either the hippocampus or cerebellum for technical validation of our microdissection and RNA extraction protocols. The relative signal intensities of two such genes, IQGAP2 and PCP2 are show; the mean log signal intensity values of the microarray probes for each of these genes is plotted for each sample type and time of sacrifice. The signal intensity threshold was set at 10 signal intensity units. IQGAP2 has been reported to be highly expressed in the CA, and PCP2 in the cerebellum and accordingly, the intensity of the array probes for IQGAP2 and PCP2 were at least a log higher in CA1 or cerebellum respectively. Allen Brain Atlas in situ hybridization images are provided to further illustrate this relationship (http://mouse.brain-map.org/)..

A

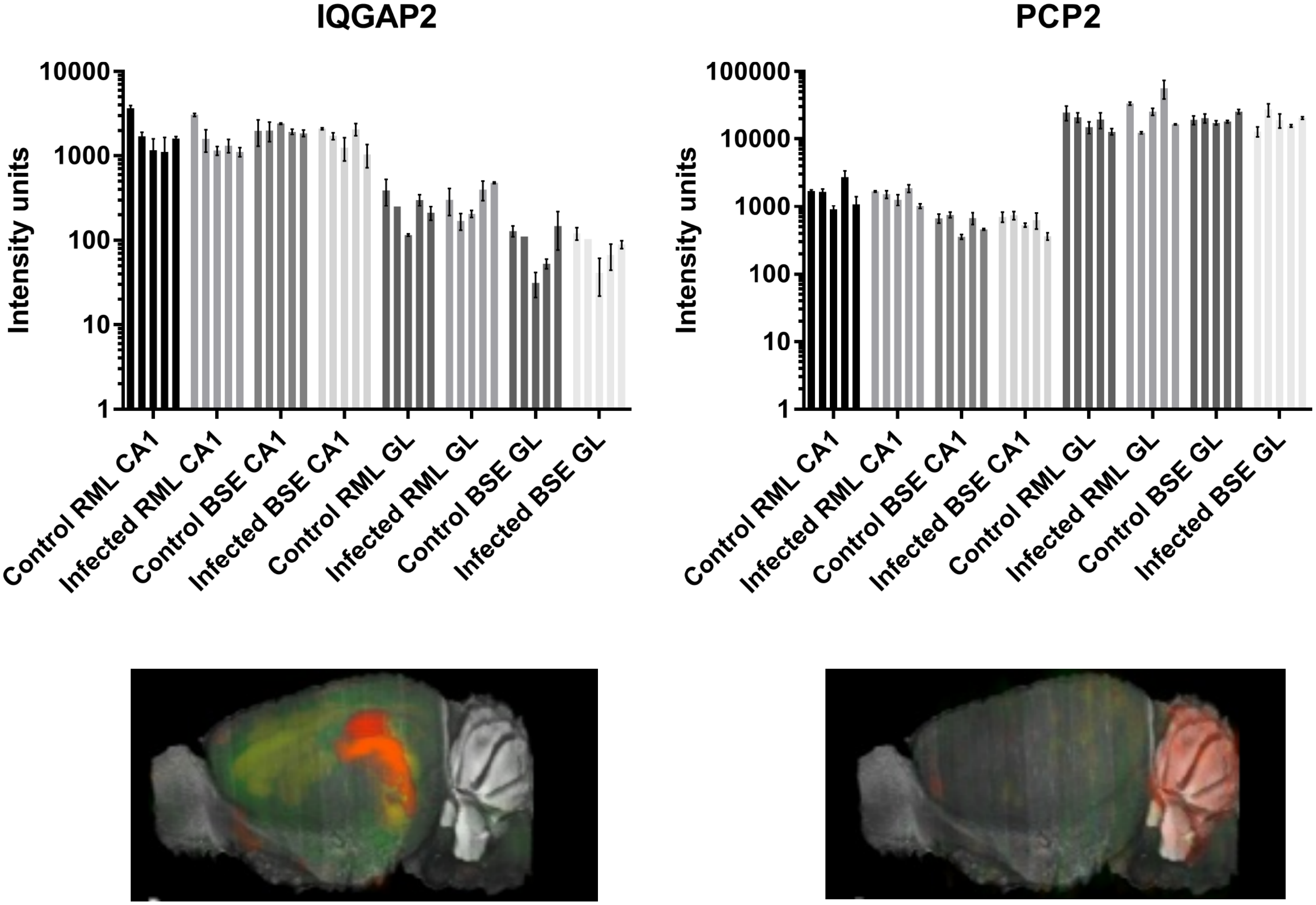

Supplemental Figure 3. Relative gene expression levels of selected neuron, microglia astrocyte and oligodendrocyte markers in CA1 during RML scrapie infection.

We chose 4 ’marker’ genes that are frequently reported in the literature as being predominantly expressed either in neurons, microglia, astrocytes or oligodendrocytes. The intensity values from the microarray data was averaged for each experimental condition and plotted. This data serves to validate the experimental approach of microdissection to select regions of tissue that are enriched with neurons.
The bars represent log signal intensity values where light gray bars indicate infected samples while dark gray bars represent control levels for each specified gene. Significance was calculated by the Student’s t-test where * represents a p-value=0.05; ** reflects a p-value=0.01 and *** stands for a p-value=0.001. The x axes represent the incubation period of disease and time points are 70, 90, 110, 130 DPI and terminal, 153-161 DPI.

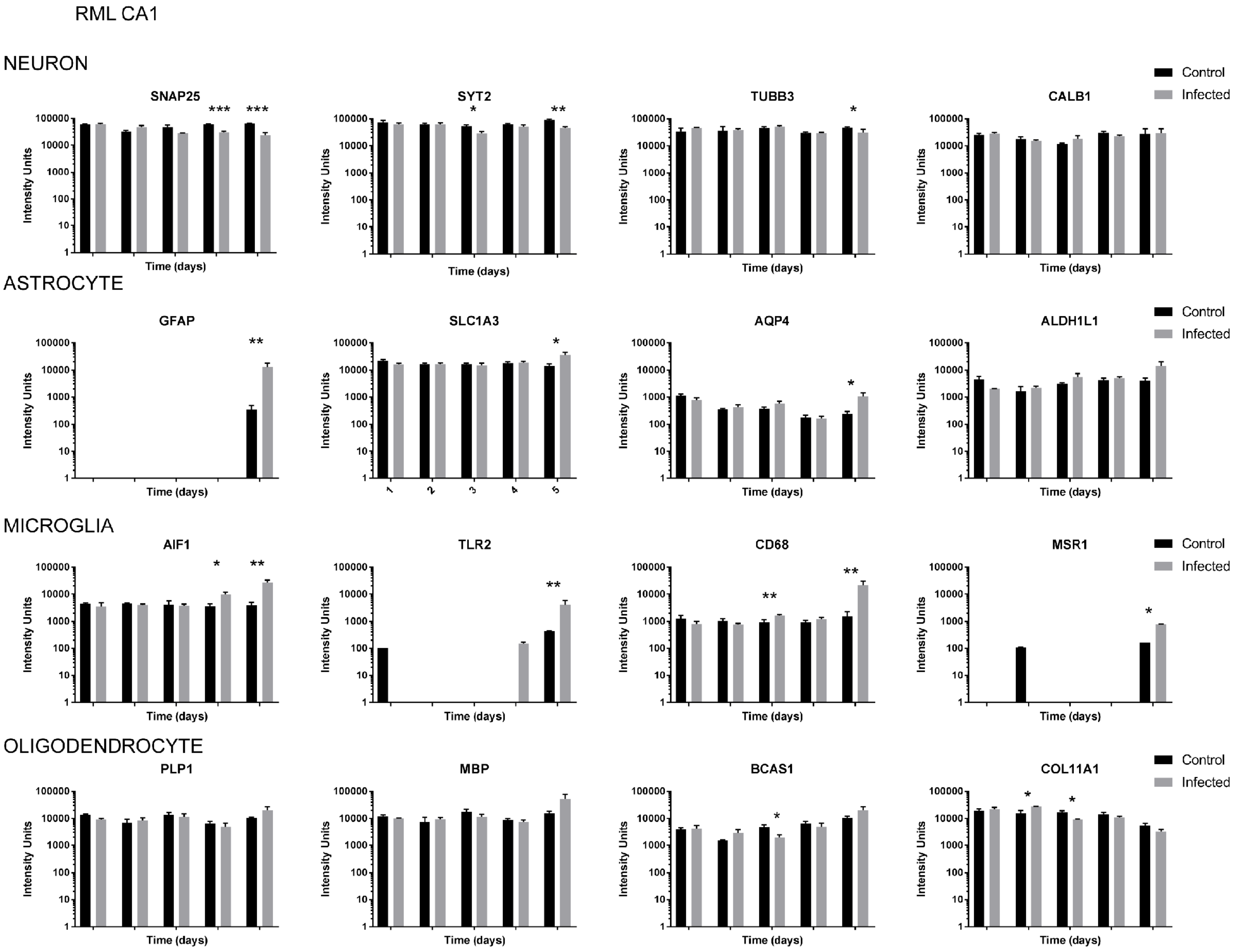

Supplemental Figure 4. Relative gene expression levels of selected neuron, microglia astrocyte and oligodendrocyte markers in cerebellum during RML scrapie disease in mice.

We chose 4 ’marker’ genes that are frequently reported in the literature as being predominantly expressed either in neurons, microglia, astrocytes or oligodendrocytes. The intensity values from control and prion infected samples microdissected from the granule layer of the cerebellum region of mice infected with RML was plotted. This data serves to validate the experimental approach of microdissection to select regions of tissue that are enriched with neurons.
The bars represent log signal intensity values where light gray bars indicate infected samples while dark gray bars represent control levels for each specified gene. Significance was calculated by the Student’s t-test where * represents a p-value=0.05; ** reflects a p-value=0.01 and *** stands for a p-value=0.001. The x axes represent the incubation period of disease and time points are 70, 90, 110, 130 DPI and terminal, 153-161 DPI.

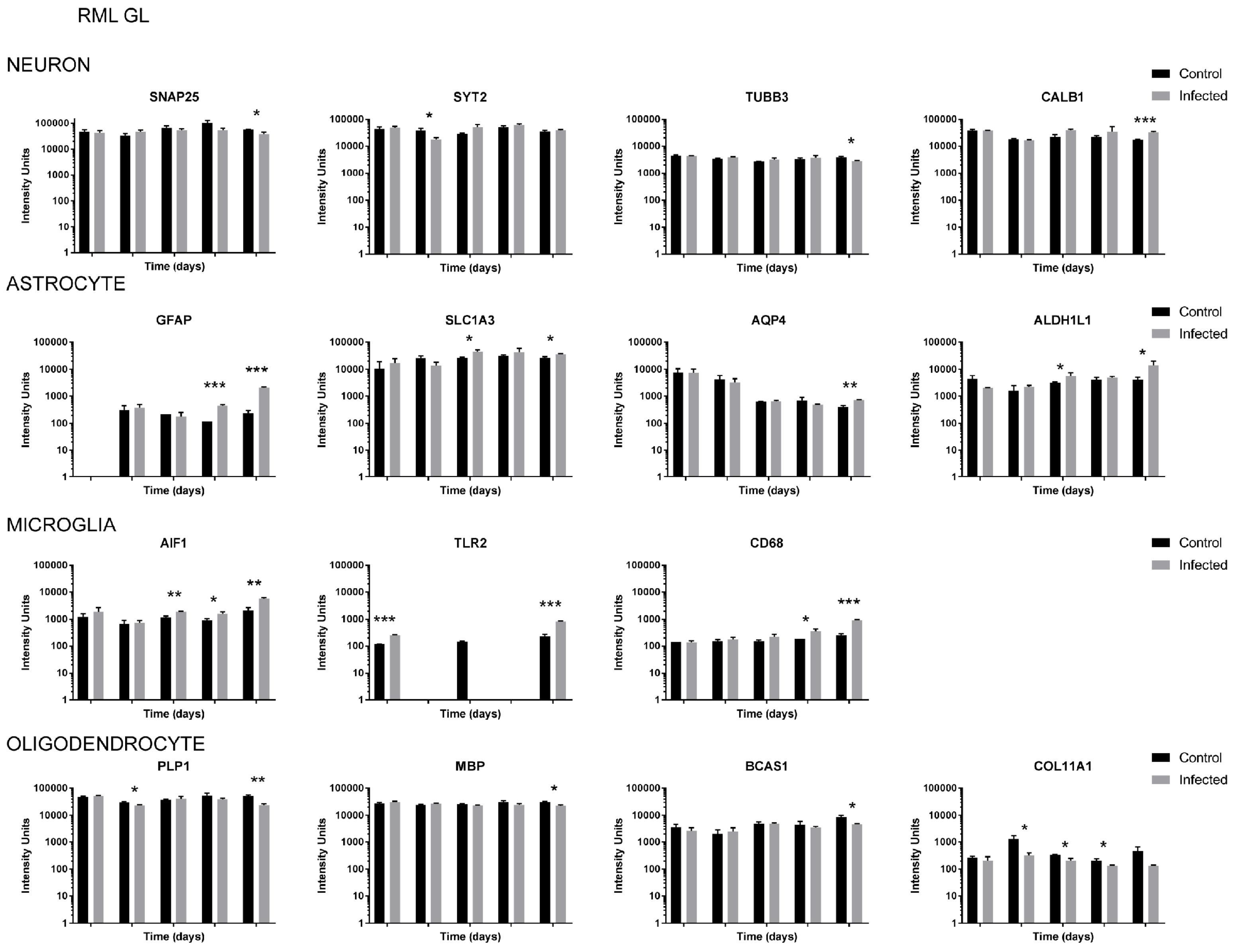

Supplemental Figure 5. Relative gene expression levels of selected neuron, microglia astrocyte and oligodendrocyte markers in CA1 during BSE disease in mice.

We chose 4 ‘marker’ genes that are frequently reported in the literature as being predominantly expressed either in neurons, microglia, astrocytes or oligodendrocytes. The intensity values from control and prion infected samples microdissected from the hippocampal CA1 region of mice infected with BSE. was plotted. This data serves to validate the experimental approach of microdissection to select regions of tissue that are enriched with neurons.

The bars represent log signal intensity values where light gray bars indicate infected samples while dark gray bars represent control levels for each specified gene. Significance was calculated by the Student‘s t-test where * represents a p-value=0.05; ** reflects a p-value=0.01 and *** stands for a p-value=0.001. The x axes represent the incubation period of disease and time points are 60, 140, 200, 300 DPI and terminal, 304-390 DPI.

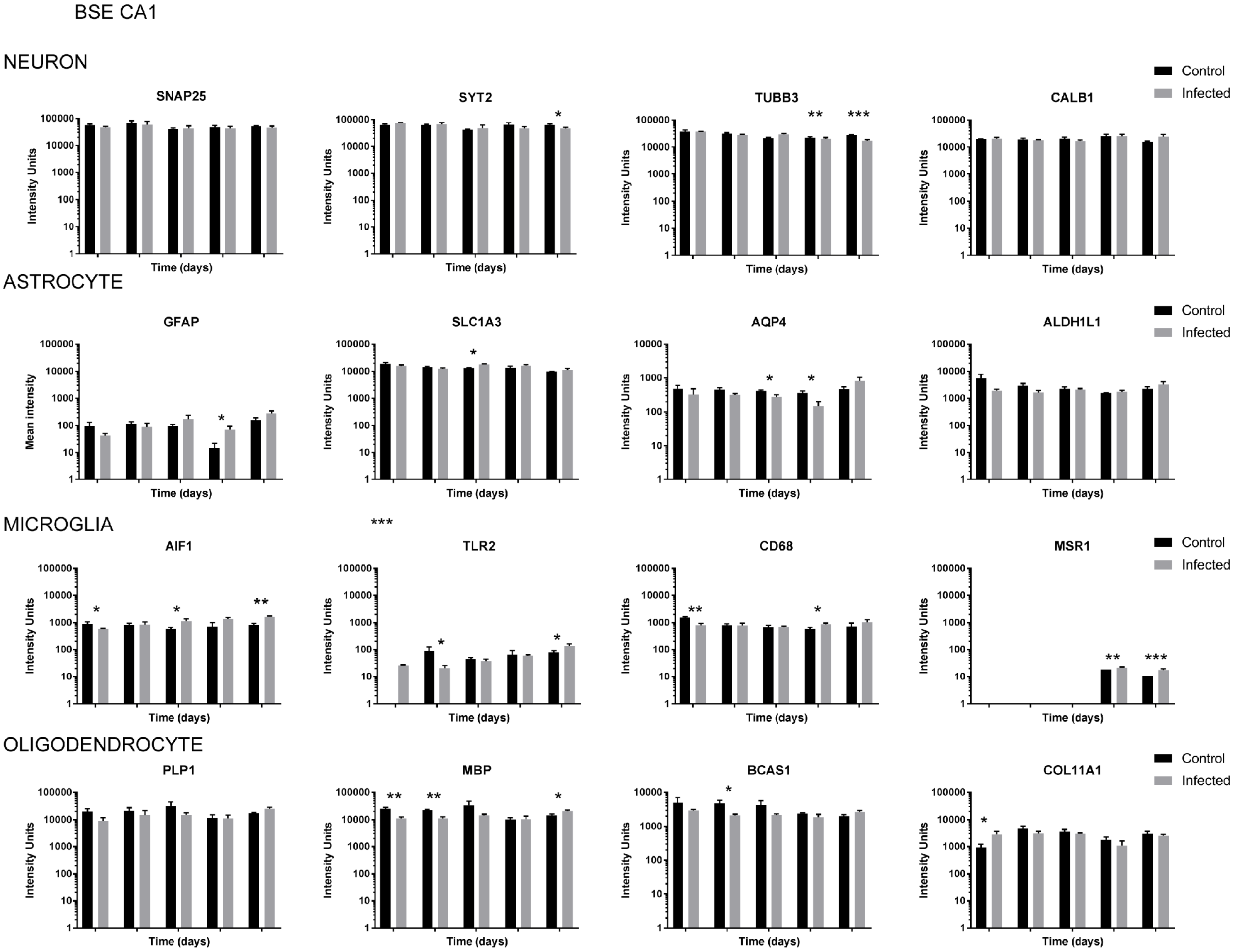

Supplemental Figure 6. Relative gene expression levels of selected neuron, microglia astrocyte and oligodendrocyte markers in cerebellum during BSE disease in mice.

We chose 4 ’marker’ genes that are frequently reported in the literature as being predominantly expressed either in neurons, microglia, astrocytes or oligodendrocytes. The intensity values from control and prion infected samples microdissected from the granule layer of the cerebellum region of mice infected with BSE was plotted. This data serves to validate the experimental approach of microdissection to select regions of tissue that are enriched with neurons.

The bars represent log signal intensity values where light gray bars indicate infected samples while dark gray bars represent control levels for each specified gene. Significance was calculated by the Student’s t-test where * represents a p-value=0.05; ** reflects a p-value=0.01 and *** stands for a p-value=0.001. The x axes represent the incubation period of disease and time points are 60, 140, 200, 300 DPI and terminal, 304-390 DPI.

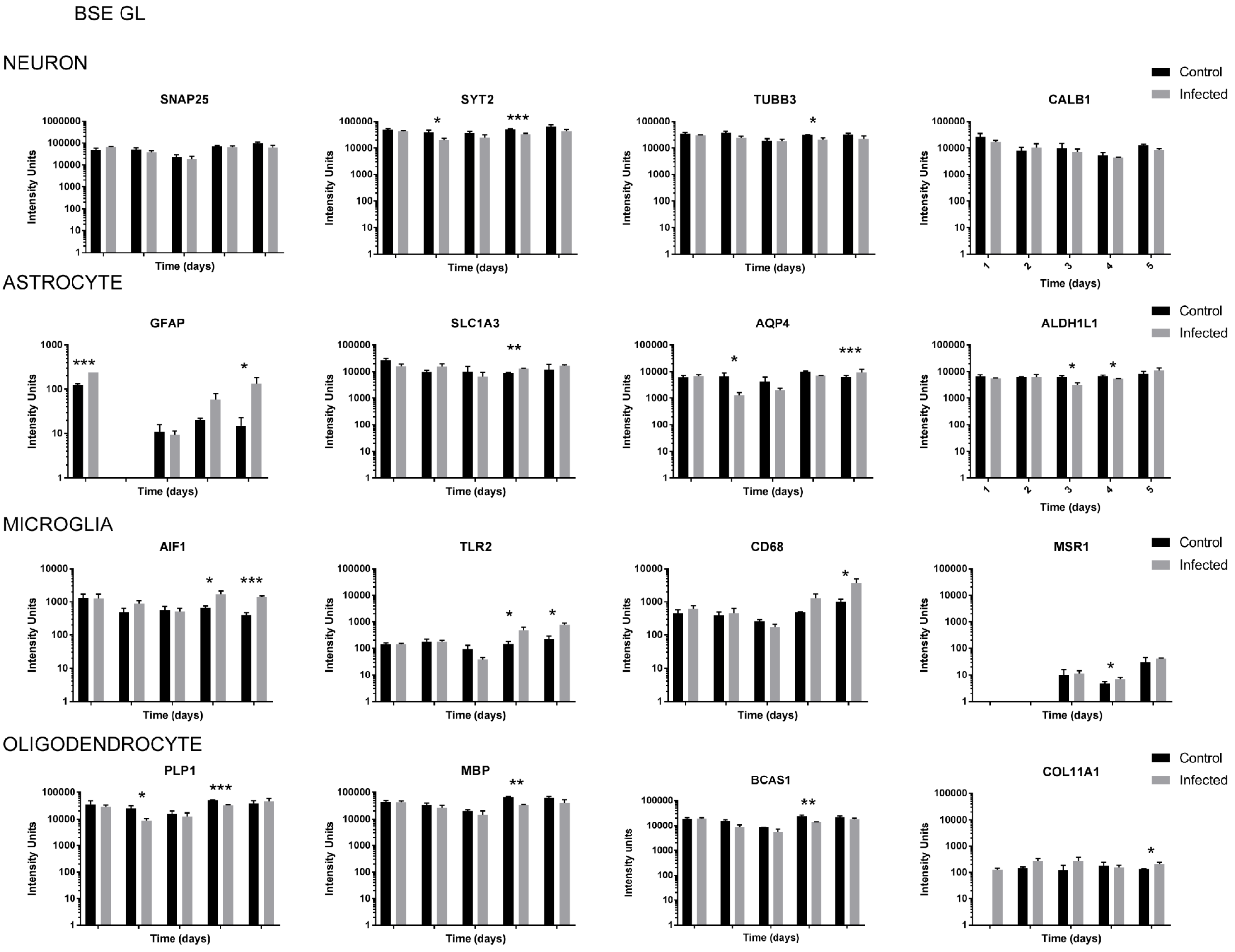

Supplemental Table 1. Genes with over 50% expression localised to astrocytes, microglia or neurons that also show altered expression profiles in microdissected CA1 and cerebellar granule region during the course of prion disease.

ASTROCYTE List

Symbol	Found in Hwang et al 2009[3] DEG	Probe Name	Gene Name
2700046G09Rik		A_52_P324656	RIKEN cDNA 2700046G09 gene
AASS		A_51_P483544	aminoadipate-semialdehyde synthase
ACTA2		A_52_P420504	actin, alpha 2, smooth muscle, aorta
ADRA2A		A_52_P67493	adrenoceptor alpha 2A
AHNAK		A_52_P263095	AHNAK nucleoprotein (desmoyokin)
ALDH1A1		A_51_P334942	aldehyde dehydrogenase 1 family, member A1
ALDH1A2		A_52_P58145	aldehyde dehydrogenase 1 family, member A2
ALDH1L1	x	A_51_P504546	aldehyde dehydrogenase 1 family, member L1
ALDOC	x	A_51_P425284	aldolase C, fructose-bisphosphate
ANGPT1	x	A_51_P338443	angiopoietin-like 4
APOD	x	A_51_P366811	apolipoprotein D
AQP4	x	A_52_P625527	aquaporin 4
ARHGAP5		A_52_P551909	Rho GTPase activating protein 5
ARMC3		A_51_P123546	armadillo repeat containing 3
ATHL1		A_51_P376789	ATH1, acid trehalase-like 1 (yeast)
ATOH8		A_51_P209736	atonal bHLH transcription factor 8
BBS2		A_51_P345196	Bardet-Biedl syndrome 2
BCAR3		A_51_P520936	breast cancer anti-estrogen resistance 3
BTBD7		A_51_P330658	BTB (POZ) domain containing 7
C1orf198		A_52_P434556	chromosome 1 open reading frame 198
C1QL3		A_51_P367977	complement component 1, q subcomponent-like 3
C1QTNF5		A_51_P373911	C1q and tumor necrosis factor related protein 5
C1R		A_51_P384318	complement component 1, r subcomponent
C2orf40		A_51_P404463	chromosome 2 open reading frame 40
C4orf19		A_51_P483617	chromosome 4 open reading frame 19
C4orf22		A_52_P418515	chromosome 4 open reading frame 22
CALML4		A_52_P367760	calmodulin-like 4
CAP2		A_52_P148069	CAP, adenylate cyclase-associated protein, 2 (yeast)
CAPN3	x	A_51_P423859	calpain 3
Casp12	x	A_51_P487518	caspase 12
CAV2		A_51_P279552	caveolin 2
CCDC102A		A_51_P138895	coiled-coil domain containing 102A
CCDC103		A_52_P405200	coiled-coil domain containing 103
CCDC113		A_51_P410576	coiled-coil domain containing 113
CCDC78		A_52_P446816	coiled-coil domain containing 78
CCDC80	x	A_51_P248786	coiled-coil domain containing 80
CCDC96		A_51_P405129	coiled-coil domain containing 96
CCPG1		A_52_P585233	cell cycle progression 1
CD164		A_52_P220090	CD164 antigen
CD44	x	A_52_P523459	CD44 antigen
CD59		A_52_P61691	CD59b antigen
CDC42EP4		A_51_P323451	CDC42 effector protein (Rho GTPase binding) 4
CECR2		A_52_P260468	cat eye syndrome chromosome region, candidate 2
CEP112		A_52_P380114	centrosomal protein 112kDa
CFI		A_51_P496905	complement factor I
CGNL1		A_51_P361678	cingulin-like 1
CHRDL1		A_52_P674808	chordin-like 1
CNTF		A_51_P378967	ciliary neurotrophic factor
COL3A1		A_51_P515605	collagen, type III, alpha 1
COL4A1		A_51_P124254	collagen, type IV, alpha 1
COL4A2		A_52_P496142	collagen, type IV, alpha 2
COL4A6		A_52_P516409	collagen, type IV, alpha 6

COL6A1		A_51_P474496	collagen, type VI, alpha 1
COL8A2		A_52_P302544	collagen, type VIII, alpha 2
COPZ2		A_51_P230904	coatomer protein complex, subunit zeta 2
CP	x	A_52_P597002	ceruloplasmin (ferroxidase)
CPNE2		A_51_P261059	copine II
CRB2		A_51_P300618	crumbs family member 2
CRTC3		A_51_P479598	CREB regulated transcription coactivator 3
CTGF		A_51_P157042	connective tissue growth factor
CYBRD1	x	A_52_P124734	cytochrome b reductase 1
CYP1B1		A_51_P255456	cytochrome P450, family 1, subfamily B, polypeptide 1
Cyp2d22		A_52_P478420	cytochrome P450, family 2, subfamily d, polypeptide 22
CYP39A1		A_52_P557265	cytochrome P450, family 39, subfamily A, polypeptide 1
DAG1		A_52_P419118	dystroglycan 1 (dystrophin-associated glycoprotein 1)
DCN		A_51_P334104	decorin
DDIT4L		A_52_P311853	DNA-damage-inducible transcript 4-like
DLK1		A_51_P135618	delta-like 1 homolog (Drosophila)
DMRTA2		A_51_P110081	DMRT-like family A2
DNAJC3		A_52_P264902	DnaJ (Hsp40) homolog, subfamily C, member 3
DNALI1		A_52_P416046	dynein, axonemal, light intermediate chain 1
DRP2		A_52_P92161	dystrophin related protein 2
DTNA		A_52_P108607	dystrobrevin, alpha
DUSP14		A_51_P322677	dual specificity phosphatase 14
DYNC2LI1		A_51_P472481	dynein, cytoplasmic 2, light intermediate chain 1
DYNLRB2		A_51_P227962	dynein, light chain, roadblock-type 2
DYSF		A_52_P233801	dysferlin
DYX1C1		A_51_P332264	dyslexia susceptibility 1 candidate 1
DZIP1L		A_51_P199618	DAZ interacting zinc finger protein 1-like
ECE2		A_52_P271572	endothelin converting enzyme 2
EFEMP1	x	A_51_P337412	EGF containing fibulin-like extracellular matrix protein 1
EGLN3		A_52_P387009	egl-9 family hypoxia-inducible factor 3
EMP1	x	A_52_P120037	epithelial membrane protein 1
ENTPD2	x	A_51_P112932	ectonucleoside triphosphate diphosphohydrolase 2
EVA1C		A_52_P6070	eva-1 homolog C (C. elegans)
F2R		A_52_P344290	coagulation factor II (thrombin) receptor
FAM71E1		A_51_P323531	family with sequence similarity 71, member E1
FAM92B		A_52_P26626	family with sequence similarity 92, member B
FAS	x	A_51_P345393	Fas cell surface death receptor
FBLN1		A_52_P588483	fibulin 1
FBLN5		A_51_P186703	fibulin 5
FBLN7		A_52_P559817	fibulin 7
FBXL7		A_52_P290434	F-box and leucine-rich repeat protein 7
FBXO30		A_51_P223036	F-box protein 30
FGF1		A_51_P148828	fibroblast growth factor 1 (acidic)
FGL1		A_51_P275905	fibrinogen-like 1
FLNB		A_51_P446856	filamin B, beta
FOXC2		A_51_P196444	forkhead box C2
FZD2		A_51_P404077	frizzled class receptor 2
FZD6		A_52_P217710	frizzled class receptor 6
GAREM		A_51_P271439	GRB2 associated, regulator of MAPK1
GCHFR		A_51_P483159	GTP cyclohydrolase I feedback regulator
GFAP	x	A_52_P52303	glial fibrillary acidic protein
GGCX		A_52_P110812	gamma-glutamyl carboxylase
GJB2		A_52_P382886	gap junction protein, beta 2, 26kDa
GLB1L		A_52_P504715	galactosidase, beta 1-like
GLI3		A_51_P171288	GLI family zinc finger 3
GLIPR2	x	A_51_P517843	GLI pathogenesis-related 2
Glycam1		A_52_P602847	glycosylation dependent cell adhesion molecule 1
GNA14		A_51_P110323	guanine nucleotide binding protein (G protein), alpha 14
GPR34	x	A_52_P220275	G protein-coupled receptor 34
GPRC5B		A_51_P173709	G protein-coupled receptor, class C, group 5, member B
HDAC8		A_52_P464062	histone deacetylase 8
HDC		A_51_P254656	histidine decarboxylase
HIPK1		A_52_P268687	homeodomain interacting protein kinase 1

HSPA2		A_51_P453149	heat shock 70kDa protein 2
HSPB1	x	A_52_P404533	heat shock 27kDa protein 1
HSPB2		A_52_P222624	heat shock 27kDa protein 2
HSPB6	x	A_51_P322022	heat shock protein, alpha-crystallin-related, B6
HSPB8	x	A_51_P464387	heat shock 22kDa protein 8
ID1		A_51_P385786	inhibitor of DNA binding 1, dominant negative helix-loop-helix protein
ID3	x	A_51_P380178	inhibitor of DNA binding 3, dominant negative helix-loop-helix protein
IFT74		A_52_P136966	intraflagellar transport 74
IGF2		A_51_P516826	insulin-like growth factor 2
IGFBP2		A_51_P423308	insulin-like growth factor binding protein 2, 36kDa
IGFBP5		A_52_P281702	insulin-like growth factor binding protein 5
IGFBP6		A_51_P374571	insulin-like growth factor binding protein 6
IGFBP7		A_51_P472292	insulin-like growth factor binding protein 7
IGSF1		A_51_P366157	immunoglobulin superfamily, member 1
IKZF2	x	A_52_P413515	IKAROS family zinc finger 2 (Helios)
IL17RD		A_51_P253732	interleukin 17 receptor D
IL1R2		A_51_P470079	interleukin 1 receptor, type II
IL4I1		A_52_P410449	interleukin 4 induced 1
INMT		A_51_P162162	indolethylamine N-methyltransferase
IQCG		A_51_P152550	IQ motif containing G
IQGAP2		A_52_P65496	IQ motif containing GTPase activating protein 2
ISLR		A_51_P140742	immunoglobulin superfamily containing leucine-rich repeat
ITGB4		A_52_P143287	integrin, beta 4
ITIH5		A_51_P364250	inter-alpha-trypsin inhibitor heavy chain family, member 5
KCNE5	x	A_51_P160673	potassium channel, voltage gated subfamily E regulatory beta subunit 5
KDR		A_51_P316553	kinase insert domain receptor
KLF2		A_51_P144264	Kruppel-like factor 2
KLF5		A_51_P507290	Kruppel-like factor 5
L3HYPDH		A_51_P408989	L-3-hydroxyproline dehydratase (trans-)
LAMB2		A_51_P248044	laminin, beta 2 (laminin S)
LBP		A_51_P454008	lipopolysaccharide binding protein
LIX1		A_52_P329443	limb and CNS expressed 1
LMCD1	x	A_51_P405912	LIM and cysteine-rich domains 1
LPAR4		A_52_P438036	lysophosphatidic acid receptor 4
LRRC56		A_51_P484200	leucine rich repeat containing 56
LUM		A_51_P167527	lumican
MAFF		A_52_P608322	v-maf avian musculoaponeurotic fibrosarcoma oncogene homolog F
MAMDC2		A_51_P451458	MAM domain containing 2
MDH1B		A_51_P227077	malate dehydrogenase 1B, NAD (soluble)
MEDAG		A_52_P3276	mesenteric estrogen-dependent adipogenesis
MEST		A_51_P124535	mesoderm specific transcript
MFI2		A_51_P324351	antigen p97 (melanoma associated) identified by monoclonal antibodies 133.2 and 96.5
MGST1	x	A_52_P549827	microsomal glutathione S-transferase 1
MIA		A_52_P415167	melanoma inhibitory activity
MLF1		A_51_P205106	myeloid leukemia factor 1
MME		A_52_P429723	membrane metallo-endopeptidase
MMP11		A_51_P293087	matrix metallopeptidase 11
MMP14		A_52_P304128	matrix metallopeptidase 14 (membrane-inserted)
MMP2		A_51_P341736	matrix metallopeptidase 2
MNS1		A_51_P100174	meiosis-specific nuclear structural 1
MORN1		A_51_P423825	MORN repeat containing 1
MR1		A_52_P212429	major histocompatibility complex, class I-related
MRO		A_52_P601230	maestro
Mt1	x	A_51_P294979	metallothionein 1
Mt2	x	A_51_P246317	metallothionein 2
MTMR11	x	A_52_P350537	myotubularin related protein 11
NEDD9		A_52_P491544	neural precursor cell expressed, developmentally down-regulated 9
NEXN		A_52_P367520	nexilin (F actin binding protein)
NFIA		A_52_P120680	nuclear factor I/A
NID1		A_51_P339943	nidogen 1
NPR2		A_52_P565549	natriuretic peptide receptor 2
NR2F2		A_52_P620399	nuclear receptor subfamily 2, group F, member 2

NTRK2		A_51_P516006	neurotrophic tyrosine kinase, receptor, type 2
NXN		A_52_P57013	nucleoredoxin
OGN		A_51_P295085	osteoglycin
OSBPL3		A_52_P360308	oxysterol binding protein-like 3
OSMR	x	A_51_P319460	oncostatin M receptor
PAMR1		A_51_P212592	peptidase domain containing associated with muscle regeneration 1
PARD6B		A_52_P37123	par-6 family cell polarity regulator beta
PAX6		A_51_P489935	paired box 6
PCOLCE		A_51_P371942	procollagen C-endopeptidase enhancer
PDLIM1		A_51_P461138	PDZ and LIM domain 1
PDLIM7		A_52_P21574	PDZ and LIM domain 7 (enigma)
PDP1		A_51_P452637	pyruvate dehyrogenase phosphatase catalytic subunit 1
PIK3IP1		A_51_P463428	phosphoinositide-3-kinase interacting protein 1
PLAGL1		A_52_P532456	pleiomorphic adenoma gene-like 1
PLCD4	x	A_52_P327588	phospholipase C, delta 4
Plscr2	x	A_52_P372901	phospholipid scramblase 2
PPM1K		A_51_P265219	protein phosphatase, Mg2+/Mn2+ dependent, 1K
PPP1R3C		A_52_P30451	protein phosphatase 1, regulatory subunit 3C
PRDX6	x	A_51_P496023	peroxiredoxin 6
PRKCDBP	x	A_51_P180974	protein kinase C, delta binding protein
PRRX2		A_51_P183746	paired related homeobox 2
PRSS23		A_52_P679105	protease, serine, 23
PRTG		A_51_P483639	protogenin
PTER		A_51_P251069	phosphotriesterase related
PYGB		A_51_P485810	phosphorylase, glycogen; brain
RAMP1		A_51_P348665	receptor (G protein-coupled) activity modifying protein 1
RAPGEF3	x	A_51_P116906	Rap guanine nucleotide exchange factor (GEF) 3
RARRES2		A_52_P466147	retinoic acid receptor responder (tazarotene induced) 2
RASL11A		A_51_P340699	RAS-like, family 11, member A
RBP1	x	A_51_P423484	retinol binding protein 1, cellular
RDH10		A_52_P59318	retinol dehydrogenase 10 (all-trans)
RDH5		A_51_P479618	retinol dehydrogenase 5 (11-cis/9-cis)
RGS20		A_51_P424690	regulator of G-protein signaling 20
RHOD		A_51_P452953	ras homolog family member D
RHOJ	x	A_52_P592466	ras homolog family member J
RIBC1		A_51_P217706	RIB43A domain with coiled-coils 1
S100A6	x	A_51_P281089	S100 calcium binding protein A6
SCNN1A		A_51_P213691	sodium channel, non voltage gated 1 alpha subunit
SERPINF1		A_51_P517075	serpin peptidase inhibitor, clade F (alpha-2 antiplasmin, pigment epithelium derived factor), member 1
SERPING1	x	A_51_P376238	serpin peptidase inhibitor, clade G (C1 inhibitor), member 1
SESN3		A_51_P246773	sestrin 3
SFT2D2		A_51_P103780	SFT2 domain containing 2
SLC12A8		A_51_P476618	solute carrier family 12, member 8
SLC13A4		A_51_P514319	solute carrier family 13 (sodium/sulfate symporter), member 4
SLC25A18	x	A_51_P507664	solute carrier family 25 (glutamate carrier), member 18
SLC2A4		A_51_P217498	solute carrier family 2 (facilitated glucose transporter), member 4
SLC4A4		A_52_P457567	solute carrier family 4 (sodium bicarbonate cotransporter), member 4
SLC6A13		A_51_P438083	solute carrier family 6 (neurotransmitter transporter), member 13
SLCO2B1	x	A_52_P255782	solute carrier organic anion transporter family, member 2B1
SMAD3		A_52_P352277	SMAD family member 3
SOCS3	x	A_51_P474459	suppressor of cytokine signaling 3
SORBS1		A_52_P652999	sorbin and SH3 domain containing 1
SOX9	x	A_52_P577484	SRY (sex determining region Y)-box 9
SPARC		A_51_P431087	secreted protein, acidic, cysteine-rich (osteonectin)
SPATA1		A_51_P374549	spermatogenesis associated 1
SPATS2L		A_52_P538470	spermatogenesis associated, serine-rich 2-like
STEAP2		A_52_P129456	STEAP family member 2, metalloreductase
STK36		A_51_P487157	serine/threonine kinase 36
STXBP4		A_52_P835711	syntaxin binding protein 4
SULF1	x	A_51_P142744	sulfatase 1
Sult1a1		A_51_P321341	sulfotransferase family 1A, phenol-preferring, member 1
SYNPO	x	A_51_P123676	synaptopodin

SYNPO2		A_51_P119776	synaptopodin 2
TAGLN	x	A_52_P517683	transgelin
TEKT1		A_51_P328890	tektin 1
TEX9		A_52_P242293	testis expressed 9
TFCP2L1	x	A_52_P175376	transcription factor CP2-like 1
TGIF2	x	A_51_P456657	TGFB-induced factor homeobox 2
TGM2	x	A_52_P220879	transglutaminase 2
TIMP2	x	A_52_P540219	TIMP metallopeptidase inhibitor 2
TM4SF1	x	A_51_P240614	transmembrane 4 L six family member 1
TMEM132B		A_52_P38908	transmembrane protein 132B
TMEM53		A_51_P441837	transmembrane protein 53
TPBG		A_52_P591017	trophoblast glycoprotein
Tpm2		A_52_P418477	tropomyosin 2, beta
TPPP3	x	A_51_P246924	tubulin polymerization-promoting protein family member 3
TST		A_51_P430082	thiosulfate sulfurtransferase (rhodanese)
VANGL1		A_52_P449417	VANGL planar cell polarity protein 1
VCAM1	x	A_51_P210956	vascular cell adhesion molecule 1
VIM	x	A_51_P392687	vimentin
WDPCP		A_52_P129443	WD repeat containing planar cell polarity effector
WNT5A		A_52_P250865	wingless-type MMTV integration site family, member 5A
WNT9A		A_51_P375453	wingless-type MMTV integration site family, member 9A
WWTR1		A_51_P502608	WW domain containing transcription regulator 1
ZBTB20		A_52_P54169	zinc finger and BTB domain containing 20
ZFYVE21		A_51_P396917	zinc finger, FYVE domain containing 21
ZNF395		A_52_P632691	zinc finger protein 395
ZNF521		A_51_P214985	zinc finger protein 521

MICROGLIA list

Symbol	Hwang DEG	Agilent	Entrez Gene Name
ABCC1		A_52_P491507	ATP-binding cassette, sub-family C (CFTR/MRP), member 1
ACACB		A_51_P239236	acetyl-Coenzyme A carboxylase beta
ACVRL1		A_52_P279329	activin A receptor, type II-like 1
ADA		A_51_P430423	adenosine deaminase
ADAM33		A_52_P333567	a disintegrin and metallopeptidase domain 33
ADAM8		A_51_P319917	a disintegrin and metallopeptidase domain 8
ADCY7	x	A_52_P586141	adenylate cyclase 7
AFP	x	A_51_P510891	alpha fetoprotein
AIF1	x	A_52_P2754	allograft inflammatory factor 1
AKAP13		A_51_P452702	A kinase (PRKA) anchor protein 13
ANGPT2	x	A_51_P201982	angiopoietin 2
ANTXR1	x	A_51_P407248	anthrax toxin receptor 2
ANXA3	x	A_51_P283590	annexin A1
ANXA4	x	A_51_P256342	annexin A4
APBB1IP		A_51_P391904	amyloid beta (A4) precursor protein-binding, family B, member 1 interacting protein
APOBEC1		A_51_P160754	apolipoprotein B mRNA editing enzyme, catalytic polypeptide 1
ARAP3		A_52_P195018	ArfGAP with RhoGAP domain, ankyrin repeat and PH domain 3
ARHGAP25	x	A_51_P248629	Rho GTPase activating protein 25
ARHGAP30		A_51_P291227	Rho GTPase activating protein 30
ARL5C		A_52_P203691	ADP-ribosylation factor-like 5C
B2M	x	A_51_P129006	beta-2 microglobulin
B4GALT1		A_52_P103391	UDP-Gal:betaGlcNAc beta 1,4- galactosyltransferase, polypeptide 1
BCL2A1	x	A_51_P494241	B-cell leukemia/lymphoma 2 related protein A1b
BIRC5		A_51_P230103	baculoviral IAP repeat-containing 5
BLNK	x	A_51_P279841	B-cell linker
BLVRA		A_51_P363103	biliverdin reductase A
BMP2K	x	A_51_P337944	predicted gene 4521; BMP2 inducible kinase
BTK		A_51_P145883	Bruton agammaglobulinemia tyrosine kinase
C10orf11		A_51_P346874	RIKEN cDNA 1700112E06 gene
C10orf54	x	A_51_P372992	RIKEN cDNA 4632428N05 gene
C1QA	x	A_51_P181451	complement component 1, q subcomponent, alpha polypeptide

C1QC	x	A_51_P102789	complement component 1, q subcomponent, C chain
CA13		A_51_P153765	carbonic anhydrase 13
CAPG	x	A_51_P273921	capping protein (actin filament), gelsolin-like
CASP1	x	A_51_P142861	caspase 1
CASP4		A_51_P511787	caspase 4, apoptosis-related cysteine peptidase
CASP8	x	A_51_P247799	caspase 8
CAST		A_51_P519295	calpastatin
CCDC88B		A_52_P322141	coiled-coil domain containing 88B
CCL2	x	A_52_P249514	chemokine (C-C motif) ligand 12; similar to monocyte chemoattractant protein-5
Ccl2	x	A_51_P286737	chemokine (C-C motif) ligand 2
CCL3L3	x	A_51_P140710	chemokine (C-C motif) ligand 3
CCL4		A_51_P509573	chemokine (C-C motif) ligand 4
CCL5	x	A_51_P485312	chemokine (C-C motif) ligand 5
Ccl6	x	A_51_P460954	chemokine (C-C motif) ligand 6
Ccl9	x	A_51_P185660	chemokine (C-C motif) ligand 9
CCRL2		A_51_P232682	chemokine (C-C motif) receptor-like 2
CD14	x	A_51_P172853	CD14 antigen
CD300A		A_52_P492142	CD300A antigen
CD300C	x	A_51_P484020	cDNA sequence AF251705
CD300LF		A_52_P422494	CD300 antigen like family member F
CD48	x	A_51_P362029	CD48 antigen
Cd52	x	A_51_P151126	CD52 antigen
CD53	x	A_51_P337675	CD53 antigen
CD68	x	A_51_P120470	CD68 antigen
CD74	x	A_51_P284608	CD74 antigen (invariant polypeptide of major histocompatibility complex, class II antigen-associated)
CD84	x	A_51_P369550	CD84 antigen
CD86	x	A_51_P174723	CD86 antigen
CDT1		A_51_P133612	chromatin licensing and DNA replication factor 1
CEBPA	x	A_52_P168567	CCAAT/enhancer binding protein (C/EBP), alpha
CEBPB	x	A_52_P63553	CCAAT/enhancer binding protein (C/EBP), beta
CELSR1		A_51_P440743	cadherin, EGF LAG seven-pass G-type receptor 1 (flamingo homolog, Drosophila)
CFLAR		A_51_P326091	CASP8 and FADD-like apoptosis regulator pseudogene; CASP8 and FADD-like apoptosis regulator
CH25H	x	A_51_P112966	cholesterol 25-hydroxylase
CKLF	x	A_51_P394715	chemokine-like factor
Clec2d		A_51_P324690	C-type lectin domain family 2, member d
Clec4a3	x	A_51_P147274	C-type lectin domain family 4, member a3
CLEC5A	x	A_52_P218058	C-type lectin domain family 5, member a
CLEC7A	x	A_51_P246653	C-type lectin domain family 7, member a
CLIC1	x	A_51_P357341	chloride intracellular channel 1
CMTM7	x	A_51_P377833	CKLF-like MARVEL transmembrane domain containing 7
CSF1R	x	A_52_P602091	colony stimulating factor 1 receptor
CSF2RB	x	A_51_P146753	colony stimulating factor 2 receptor, beta 2, low-affinity (granulocyte-macrophage)
CSF3R	x	A_51_P515639	predicted gene 4223; similar to Csf3r protein; colony stimulating factor 3 receptor (granulocyte)
CST7	x	A_51_P137419	cystatin F (leukocystatin)
CSTA	x	A_51_P384894	cystatin A
CSTB	x	A_52_P7400	cystatin B
Ctla2a/Ctla2b	x	A_51_P180747	cytotoxic T lymphocyte-associated protein 2 alpha
CTSA	x	A_52_P672803	cathepsin A
CTSB	x	A_51_P465148	cathepsin B
CTSC	x	A_52_P195839	cathepsin C
CTSS	x	A_51_P423981	cathepsin S
CTSZ	x	A_51_P256202	cathepsin Z
CX3CR1	x	A_51_P350853	similar to chemokine receptor CX3CR1; chemokine (C-X3-C) receptor 1
CXCL13	x	A_51_P378789	chemokine (C-X-C motif) ligand 13
CXCL16	x	A_51_P374203	chemokine (C-X-C motif) ligand 16
CYTH4	x	A_52_P271099	cytohesin 4
DENND4C		A_51_P192998	DENN/MADD domain containing 4C

DHX58	x	A_52_P223809	DEXH (Asp-Glu-X-His) box polypeptide 58
DOK1	x	A_51_P275976	docking protein 1
E2F1		A_52_P587611	E2F transcription factor 1
EBI3	x	A_51_P202633	Epstein-Barr virus induced gene 3
EIF2AK2		A_52_P559919	eukaryotic translation initiation factor 2-alpha kinase 2
EMP3	x	A_51_P446510	epithelial membrane protein 3
ENPP1		A_51_P231499	ectonucleotide pyrophosphatase/phosphodiesterase 1
EPSTI1		A_51_P376050	epithelial stromal interaction 1 (breast)
FABP4		A_51_P336830	fatty acid binding protein 4, adipocyte
FAM167B		A_52_P326657	family with sequence similarity 167, member B
FCER1G	x	A_51_P405478	Fc receptor, IgE, high affinity I, gamma polypeptide
FCGR1A	x	A_52_P47846	Fc receptor, IgG, high affinity I
FCGR2A	x	A_52_P408757	Fc receptor, IgG, low affinity III
Fcrls	x	A_51_P172168	Fc receptor-like S, scavenger receptor
FES	x	A_52_P365011	feline sarcoma oncogene
FLI1	x	A_52_P821	Friend leukemia integration 1
FLT1	x	A_52_P307922	FMS-like tyrosine kinase 1
GALNT12		A_51_P188911	UDP-N-acetyl-alpha-D-galactosamine:polypeptide N-acetylgalactosaminyltransferase 12
GALNT6	x	A_52_P111715	UDP-N-acetyl-alpha-D-galactosamine:polypeptide N-acetylgalactosaminyltransferase 6
GCNT1		A_52_P21550	glucosaminyl (N-acetyl) transferase 1, core 2; similar to glucosaminyl (N-acetyl) transferase 1, core 2
GMFG	x	A_51_P156868	glia maturation factor, gamma
GPNMB	x	A_51_P438967	glycoprotein (transmembrane) nmb
GPR183	x	A_51_P358683	G protein-coupled receptor 183
GPR65	x	A_51_P108459	G-protein coupled receptor 65
GPR84	x	A_51_P276993	G protein-coupled receptor 84
GPSM3	x	A_51_P106322	G-protein signalling modulator 3 (AGS3-like, C. elegans)
GSDMD		A_51_P172231	gasdermin D
GUSB	x	A_51_P211491	glucuronidase, beta
HAVCR2	x	A_51_P109508	hepatitis A virus cellular receptor 2
HCK	x	A_51_P499918	hemopoietic cell kinase
HCST		A_52_P170188	hematopoietic cell signal transducer
HELZ2		A_52_P321358	cDNA sequence BC006779
HLA-A	x	A_51_P190499	histocompatibility 2, K1, K region; similar to H-2K(d) antigen
HLA-DMB	x	A_51_P278868	histocompatibility 2, class II, locus Mb1
HLA-G		A_51_P469968	histocompatibility 2, M region locus 3
HPGDS	x	A_52_P536796	prostaglandin D2 synthase 2, hematopoietic
HVCN1	x	A_51_P331831	hydrogen voltage-gated channel 1
ICAM1	x	A_51_P408506	intercellular adhesion molecule 1
IFI16		A_51_P408346	interferon activated gene 204
IFI203		A_51_P237856	similar to Interferon-activatable protein 203 (Ifi-203) (Interferon-inducible protein p203)
Ifi27l2a/Ifi27l2b	x	A_51_P358233	interferon, alpha-inducible protein 27 like 2A
IFI35	x	A_51_P414889	interferon-induced protein 35
Ifi47	x	A_51_P129229	interferon gamma inducible protein 47
IFIH1	x	A_51_P387810	interferon induced with helicase C domain 1
IFIT1B	x	A_51_P327751	interferon-induced protein with tetratricopeptide repeats 1
IFIT3	x	A_51_P359570	interferon-induced protein with tetratricopeptide repeats 3
IFITM3	x	A_51_P128876	interferon induced transmembrane protein 3
Ifitm6		A_51_P294555	interferon induced transmembrane protein 6
Igtp	x	A_51_P112355	interferon gamma induced GTPase
Iigp1	x	A_51_P387239	interferon inducible GTPase 1; interferon-inducible GTPase-like
IL13RA1	x	A_51_P365189	interleukin 13 receptor, alpha 1
INPP5D	x	A_51_P337125	inositol polyphosphate-5-phosphatase D
IQGAP3		A_52_P588881	IQ motif containing GTPase activating protein 3
IRF5	x	A_51_P346668	interferon regulatory factor 5
IRF7	x	A_51_P421876	interferon regulatory factor 7
IRF8	x	A_52_P354823	interferon regulatory factor 8
IRF9	x	A_51_P127367	interferon regulatory factor 9
IRGM	x	A_51_P416295	immunity-related GTPase family M member 2
Irgm1	x	A_52_P126158	immunity-related GTPase family M member 1

ISG15		A_52_P463936	ISG15 ubiquitin-like modifier; predicted gene 9706
ISG20		A_51_P510713	interferon-stimulated protein
ITGAX	x	A_51_P303424	integrin alpha X
ITGB2	x	A_51_P262208	integrin beta 2
ITPRIPL2	x	A_51_P263407	inositol 1,4,5-triphosphate receptor interacting protein-like 1
KCNK6	x	A_51_P315890	potassium inwardly-rectifying channel, subfamily K, member 6
LAIR1	x	A_51_P503797	leukocyte-associated Ig-like receptor 1
LAPTM5	x	A_52_P13815	lysosomal-associated protein transmembrane 5
LAT2	x	A_51_P242930	linker for activation of T cells family, member 2
LCP1	x	A_52_P56751	lymphocyte cytosolic protein 1
LCP2	x	A_51_P352875	lymphocyte cytosolic protein 2
LGALS1	x	A_51_P465281	lectin, galactose binding, soluble 1
LGALS3BP	x	A_51_P359636	lectin, galactoside-binding, soluble, 3 binding protein
LGALS9B	x	A_51_P500813	lectin, galactose binding, soluble 9
LITAF	x	A_51_P267239	LPS-induced TN factor
LPXN	x	A_52_P577748	leupaxin
LRMP		A_51_P130079	
LRP1		A_52_P202045	low density lipoprotein receptor-related protein 1
Lst1	x	A_51_P205008	leukocyte specific transcript 1
LY86	x	A_51_P465350	lymphocyte antigen 86
LY96		A_51_P476601	lymphocyte antigen 96
LYN	x	A_51_P345422	Yamaguchi sarcoma viral (v-yes-1) oncogene homolog
LYZ	x	A_52_P238027	lysozyme 2
MAFB		A_52_P495869	v-maf musculoaponeurotic fibrosarcoma oncogene family, protein B (avian)
MAN2B1	x	A_51_P336161	mannosidase 2, alpha B1
MAPKAPK2		A_51_P118902	MAP kinase-activated protein kinase 2
MPEG1	x	A_51_P390538	macrophage expressed gene 1
MS4A6A	x	A_51_P149714	membrane-spanning 4-domains, subfamily A, member 6D
Ms4a6b	x	A_51_P422300	membrane-spanning 4-domains, subfamily A, member 6B
MS4A7	x	A_51_P277216	membrane-spanning 4-domains, subfamily A, member 7
MSR1		A_51_P404846	macrophage scavenger receptor 1
MYO1F	x	A_52_P113190	myosin IF
MYO1G		A_51_P333253	myosin IG
MYOF		A_51_P478353	myoferlin
NAIP	x	A_51_P265026	NLR family, apoptosis inhibitory protein 1, related sequence 1; NLR family, apoptosis inhibitory protein 2
Naip1 (includes others)	x	A_52_P300786	NLR family, apoptosis inhibitory protein 5
NCF2	x	A_51_P402909	neutrophil cytosolic factor 2; neutrophil cytosolic factor 2 related sequence
NCF4	x	A_51_P377452	neutrophil cytosolic factor 4
NCKAP1L	x	A_51_P119429	NCK associated protein 1 like
NFKBID		A_51_P322954	nuclear factor of kappa light polypeptide gene enhancer in B-cells inhibitor, delta
NQO2		A_51_P229536	NAD(P)H dehydrogenase, quinone 2
Oas1d (includes others)	x	A_51_P428529	2'-5' oligoadenylate synthetase 1C
Oasl2	x	A_51_P387123	2'-5' oligoadenylate synthetase-like 2
OLFML3	x	A_51_P191782	olfactomedin-like 3
P2RX7	x	A_52_P393314	purinergic receptor P2X, ligand-gated ion channel, 7
P2RY6	x	A_51_P105124	pyrimidinergic receptor P2Y, G-protein coupled, 6
PARP14	x	A_51_P514712	poly (ADP-ribose) polymerase family, member 14
PARP9	x	A_51_P218984	poly (ADP-ribose) polymerase family, member 9
PARVG		A_52_P531651	parvin, gamma
PDE1C		A_52_P208222	phosphodiesterase 1C
PF4		A_51_P441426	platelet factor 4
Pgap2		A_51_P226740	post-GPI attachment to proteins 2
PIK3CD		A_52_P99848	phosphatidylinositol 3-kinase catalytic delta polypeptide; RIKEN cDNA 2610208K16 gene
PLAUR		A_51_P112405	plasminogen activator, urokinase receptor
PLCG2		A_51_P279163	phospholipase C, gamma 2
PLD4	x	A_52_P591153	phospholipase D family, member 4

PLEKHO2		A_51_P293982	pleckstrin homology domain containing, family O member 2
PLIN2		A_51_P258150	adipose differentiation related protein
PON3	x	A_52_P662226	paraoxonase 3
PPFIBP2		A_51_P230439	protein tyrosine phosphatase, receptor-type, F interacting protein, binding protein 2
PSMA8		A_51_P213666	proteasome (prosome, macropain) subunit, alpha type, 8
PSMB8	x	A_51_P345366	proteasome (prosome, macropain) subunit, beta type 8 (large multifunctional peptidase 7)
PSMB9	x	A_51_P369803	proteasome (prosome, macropain) subunit, beta type 9 (large multifunctional peptidase 2)
PTGES		A_51_P312327	prostaglandin E synthase
PTPN18	x	A_51_P236118	protein tyrosine phosphatase, non-receptor type 18
PTPN6	x	A_52_P114722	protein tyrosine phosphatase, non-receptor type 6
PTPRC	x	A_52_P677117	protein tyrosine phosphatase, receptor type, C
PYCARD	x	A_51_P438820	PYD and CARD domain containing
RAB32	x	A_51_P293688	RAB32, member RAS oncogene family
RAC2		A_51_P121891	RAS-related C3 botulinum substrate 2
RASGRP3		A_52_P198435	RAS, guanyl releasing protein 3
RASSF4		A_52_P650307	Ras association (RalGDS/AF-6) domain family member 4
RCSD1		A_51_P371091	RCSD domain containing 1
RENBP	x	A_51_P123765	renin binding protein
RGS10	x	A_51_P218774	regulator of G-protein signalling 10
RHOH		A_51_P489289	ras homolog gene family, member H
RSAD2	x	A_52_P670026	radical S-adenosyl methionine domain containing 2
RTP4	x	A_51_P304170	receptor transporter protein 4
S100A4	x	A_51_P105078	hippocampus abundant transcript-like 1; S100 calcium binding protein A4
SAMSN1	x	A_52_P646832	SAM domain, SH3 domain and nuclear localization signals, 1
SASH3	x	A_51_P447866	SAM and SH3 domain containing 3
SEPP1		A_51_P470328	selenoprotein P, plasma, 1
SKAP2	x	A_51_P243914	src family associated phosphoprotein 2
SLAMF9	x	A_51_P246066	SLAM family member 9
SLC11A1	x	A_51_P186476	solute carrier family 11 (proton-coupled divalent metal ion transporters), member 1
SLC11A2		A_51_P388737	solute carrier family 11 (proton-coupled divalent metal ion transporters), member 2
SLC15A3	x	A_52_P467389	solute carrier family 15, member 3
SLC25A18	x	A_51_P507664	solute carrier family 25 (mitochondrial carrier, phosphate carrier), member 24
SLC37A2		A_51_P259774	solute carrier family 37 (glycerol-3-phosphate transporter), member 2
SLC7A7	x	A_51_P472249	solute carrier family 7 (cationic amino acid transporter, y+ system), member 7
Slfn2	x	A_51_P423578	schlafen 2
SLFN5		A_51_P364694	schlafen 5; hypothetical protein LOC100047131
SOAT1	x	A_52_P651833	sterol O-acyltransferase 1
SP110		A_52_P512201	predicted gene 15753; Sp110 nuclear body protein
SPP1	x	A_51_P358765	secreted phosphoprotein 1
Srgn	x	A_51_P193019	serglycin
STAT1	x	A_52_P70255	signal transducer and activator of transcription 1
TBXAS1	x	A_51_P448664	thromboxane A synthase 1, platelet
TGFB1	x	A_51_P390715	transforming growth factor, beta 1
TGFBR2	x	A_51_P450573	transforming growth factor, beta receptor II
TIFA	x	A_51_P249989	TRAF-interacting protein with forkhead-associated domain; similar to Traf2 binding protein
TLN1	x	A_51_P446865	talin 1
TLR1	x	A_51_P495581	toll-like receptor 1
TLR2	x	A_51_P452629	toll-like receptor 2
TLR7	x	A_51_P371710	toll-like receptor 7
TMEM140		A_52_P463977	transmembrane protein 140
TMEM173	x	A_51_P240801	transmembrane protein 173
TMEM37		A_51_P401987	transmembrane protein 37
TNFAIP8L2	x	A_51_P150678	tumor necrosis factor, alpha-induced protein 8-like 2
TNFRSF1A	x	A_52_P192426	tumor necrosis factor receptor superfamily, member 1b

TNNI2	x	A_51_P355122	troponin I, skeletal, fast 2
TNXB		A_51_P505530	tenascin XB
TOR3A	x	A_51_P198179	torsin family 3, member A
TREM2	x	A_51_P269546	triggering receptor expressed on myeloid cells 2
TRIM25	x	A_51_P212854	tripartite motif-containing 25
Trim30a/Trim30d	x	A_51_P275454	tripartite motif-containing 30
TSPO	x	A_51_P222657	translocator protein
TUBA1C		A_52_P479099	tubulin, alpha 1C; predicted gene 6682
TYROBP	x	A_51_P261517	TYRO protein tyrosine kinase binding protein
UBA7		A_51_P316816	ubiquitin-activating enzyme E1-like; RIKEN cDNA D330022A01 gene
UCP2	x	A_51_P297105	uncoupling protein 2 (mitochondrial, proton carrier)
UGT1A6	x	A_51_P308362	similar to UDP glycosyltransferase 1 family polypeptide A13
USP18	x	A_51_P164219	ubiquitin specific peptidase 18; similar to ubiquitin specific protease UBP43
VAMP8	x	A_52_P279152	vesicle-associated membrane protein 8
Wfdc17		A_52_P700056	predicted gene 11428
Xlr4a		A_51_P457244	X-linked lymphocyte-regulated 4D
ZC3HAV1		A_51_P177744	zinc finger CCCH type, antiviral 1
ZNF169		A_52_P196743	zinc finger protein 169

Neuron list

Symbol	Hwang DEG	ProbeName	Gene Name
ABCA9	x	A_52_P51503	ATP-binding cassette, sub-family A (ABC1), member 9(Abca9)
ABLIM1		A_52_P489778	actin-binding LIM protein 1
ACVR2A		A_52_P664656	activin receptor IIA(Acvr2a)
ADAMTS15		A_51_P311038	a disintegrin-like and metallopeptidase (reprolysin type) with thrombospondin type 1 motif, 15
ADCY1		A_51_P196718	adenylate cyclase 1(Adcy1)
ADD2		A_52_P260570	adducin 2 (beta)(Add2)
AGTPBP1		A_51_P519369	ATP/GTP binding protein 1
Akap9		A_52_P567697	A kinase (PRKA) anchor protein (yotiao) 9
ALDH1A3		A_52_P87843	aldehyde dehydrogenase family 1, subfamily A3(Aldh1a3)
Aldh1a7		A_51_P383399	aldehyde dehydrogenase family 1, subfamily A7(Aldh1a7)
AMPH		A_51_P460739	amphiphysin(Amph)
Ank2		A_51_P319562	ankyrin 2, brain(Ank2)
ANKRD12		A_51_P322759	ankyrin repeat domain 12; similar to Ankrd12 protein
APBA1		A_52_P429944	amyloid beta (A4) precursor protein binding, family A, member 1(Apba1)
ARHGAP26		A_52_P187820	Rho GTPase activating protein 26(Arhgap26)
ARPP21		A_52_P582732	cyclic AMP-regulated phosphoprotein, 21
ATP8A1		A_52_P385896	ATPase, aminophospholipid transporter (APLT), class I, type 8A, member 1
ATRNL1		A_52_P584293	attractin like 1
B3GALT2		A_52_P671399	UDP-Gal:betaGlcNAc beta 1,3-galactosyltransferase, polypeptide 2(B3galt2)
BDNF		A_52_P384100	brain derived neurotrophic factor
BEAN1		A_51_P111962	brain expressed, associated with Nedd4
BEND6		A_51_P280666	BEN domain containing 6
C8orf34		A_51_P172952	chromosome 8 open reading frame 34
Cabp1		A_51_P386142	calcium binding protein 1
CACNA1H		A_52_P588378	calcium channel, voltage-dependent, T type, alpha 1H subunit(Cacna1h)
CACNA2D1		A_52_P160543	calcium channel, voltage-dependent, alpha 2/delta subunit 2(Cacna2d2)
CACNA2D2		A_51_P171711	calcium channel, voltage-dependent, alpha2/delta subunit 1(Cacna2d1)
Cadm2		A_51_P442642	cell adhesion molecule 2
CADPS		A_52_P121903	Ca2+-dependent secretion activator
CADPS2		A_51_P233153	Ca2+-dependent activator protein for secretion 2
CALB2		A_51_P311436	calbindin 2(Calb2)
CALM1		A_51_P185971	calmodulin 1(Calm1)
CALN1		A_51_P199407	calneuron 1
CAMK4		A_52_P672960	calcium/calmodulin-dependent protein kinase IV
CAMTA1		A_52_P617512	calmodulin binding transcription activator 1

CASKIN1	A_51_P341926	CASK interacting protein 1
CBLN2	A_52_P233703	cerebellin 2 precursor protein
CCK	A_52_P539124	cholecystokinin(Cck)
CCSER1	A_52_P618671	coiled-coil serine-rich protein 1
CDH10	A_51_P401761	cadherin 10(Cdh10)
CDH8	A_52_P653678	cadherin 8(Cdh8)
CELF2	A_52_P499551	CUG triplet repeat, RNA binding protein 2
CHD5	A_52_P18299	chromodomain helicase DNA binding protein 5(Chd5)
CHRM3	A_52_P92072	cholinergic receptor, muscarinic 3, cardiac(Chrm3)
CISH	A_51_P470715	cytokine inducible SH2-containing protein
CLASP2	A_52_P373504	CLIP associating protein 2
CLSTN2	A_51_P370423	calsyntenin 2(Clstn2)
CLVS1	A_51_P333859	retinaldehyde binding protein 1
CNRIP1	A_51_P131687	cannabinoid receptor interacting protein 1
CNTN2	A_52_P365732	contactin 2
COX6A2	A_51_P509997	cytochrome c oxidase, subunit VI a, polypeptide 2
CPLX2	A_52_P650908	complexin 3
CPNE4	A_52_P392314	copine IV
CSRNP3	A_52_P326531	cysteine-serine-rich nuclear protein 3(Csrnp3)
CSRP2	A_51_P300337	cysteine and glycine-rich protein 2
CTNND2	A_52_P587441	catenin (cadherin associated protein), delta 2
CTTNBP2	A_52_P284243	cortactin binding protein 2
DACT1	A_51_P175567	dishevelled-binding antagonist of beta-catenin 1(Dact1)
DCLK3	A_52_P442414	doublecortin-like kinase 3
DCX	A_52_P244349	doublecortin
DLEU7	A_52_P478394	deleted in lymphocytic leukemia, 7
DLG2	A_52_P1131821	discs, large homolog 2 (Drosophila)(Dlg2)
DLGAP1	A_52_P445843	discs, large (Drosophila) homolog-associated protein 1(Dlgap1)
DMXL2	A_51_P141660	Dmx-like 2(Dmxl2)
DNAJB12	A_52_P92302	DnaJ (Hsp40) homolog, subfamily B, member 12
DNAJB5	A_52_P304264	DnaJ heat shock protein family (Hsp40) member B5(Dnajb5)
DOCK3	A_52_P739775	dedicator of cyto-kinesis 3
DOK5	A_51_P380709	docking protein 5(Dok5)
EDIL3	A_52_P325527	EGF-like repeats and discoidin I-like domains 3
EEF1A2	A_51_P485862	eukaryotic translation elongation factor 1 alpha 2(Eef1a2)
ELMOD1	A_51_P118061	ELMO domain containing 1
EPHA3	A_52_P276955	Eph receptor A3(Epha3)
EPHA4	A_52_P288177	Eph receptor A4
EPHA5	A_52_P100484	Eph receptor A5
EPHA7	A_52_P504787	Eph receptor A7
ERC2	A_51_P209903	ELKS/RAB6-interacting/CAST family member 2
FAM110D	A_51_P128463	glycine/arginine rich protein 1
FAM169A	A_52_P488623	family with sequence similarity 169, member A
FAT4	A_51_P353056	FAT tumor suppressor homolog 4 (Drosophila)
FGF12	A_52_P193161	fibroblast growth factor 12
FGF13	A_51_P409220	fibroblast growth factor 13
FGF19	A_51_P440985	fibroblast growth factor 19
FGF9	A_51_P497152	fibroblast growth factor 9
FOXO6	A_52_P589568	forkhead box O6
FSTL5	A_51_P296015	follistatin-like 5
FUT9	A_51_P417758	fucosyltransferase 8
FZD9	A_51_P511015	frizzled homolog 9 (Drosophila)
GABRA1	A_52_P259184	gamma-aminobutyric acid (GABA) A receptor, subunit alpha 1(Gabra1)
GABRA2	A_51_P417507	gamma-aminobutyric acid (GABA) A receptor, subunit alpha 2(Gabra2)
GABRA5	A_52_P143037	gamma-aminobutyric acid (GABA) A receptor, subunit alpha 5
GABRB2	A_51_P227679	gamma-aminobutyric acid (GABA) A receptor, subunit beta 2
GABRG2	A_52_P444611	gamma-aminobutyric acid (GABA) A receptor, subunit gamma 2(Gabrg2)
GALNTL6	A_52_P385718	UDP-N-acetyl-alpha-D-galactosamine:polypeptide N-acetylgalactosaminyltransferase-like 6(Galntl6)
GAP43	A_51_P290065	growth associated protein 43(Gap43)
GARNL3	A_52_P537376	GTPase activating RANGAP domain-like 3(Garnl3)
GDA	A_51_P291950	guanine deaminase
GJD2	A_52_P538310	gap junction protein, delta 2(Gjd2)

GNAL		A_52_P227481	guanine nucleotide binding protein, alpha stimulating, olfactory type(Gnal)
GNAZ		A_52_P307761	guanine nucleotide binding protein, alpha z subunit
GNG7		A_51_P443686	guanine nucleotide binding protein (G protein), gamma 7(Gng7)
GPC5		A_52_P310398	glypican 5(Gpc5)
GPR12		A_51_P104722	G-protein coupled receptor 12
GPR88		A_51_P201873	G-protein coupled receptor 88(Gpr88)
GRIA1		A_51_P498031	glutamate receptor, ionotropic, AMPA1 (alpha 1)(Gria1)
GRIA2		A_52_P575176	glutamate receptor, ionotropic, AMPA2 (alpha 2)
GRIK1		A_51_P282609	glutamate receptor, ionotropic, kainate 1
GRIN1		A_52_P69292	glutamate receptor, ionotropic, NMDA1 (zeta 1)(Grin1)
GRIN2B		A_51_P229869	glutamate receptor, ionotropic, NMDA2B (epsilon 2)
GRM2		A_51_P494725	glutamate receptor, metabotropic 2(Grm2)
GUCY1B3		A_51_P253303	guanylate cyclase 1, soluble, beta 3
HAPLN1		A_51_P101787	hyaluronan and proteoglycan link protein 1
HBA1		A_52_P278538	hemoglobin, alpha 1
HCN1		A_52_P311313	hyperpolarization-activated, cyclic nucleotide-gated K+ 1(Hcn1)
HDAC9		A_52_P411780	histone deacetylase 9
HDGFRP3		A_52_P317230	hepatoma-derived growth factor, related protein 3(Hdgfrp3)
HECTD2		A_52_P596458	HECT domain containing 2(Hectd2)
HECTD4		A_51_P159902	HECT domain containing E3 ubiquitin protein ligase 4
HECW1		A_52_P454172	HECT, C2 and WW domain containing E3 ubiquitin protein ligase 1(Hecw1)
HOMER1		A_52_P151211	homer homolog 1 (Drosophila)
HOMER2		A_51_P352303	homer homolog 2 (Drosophila)
HPCA		A_52_P287246	hippocalcin
HPCAL4		A_51_P388819	hippocalcin-like 4
HS6ST2		A_52_P599317	heparan sulfate 6-O-sulfotransferase 2
HSPB3		A_51_P419007	heat shock protein 3
IGSF3		A_51_P327632	immunoglobulin superfamily, member 3(Igsf3)
INHBA		A_51_P239750	inhibin beta-A(Inhba)
JAKMIP2		A_52_P1188177	janus kinase and microtubule interacting protein 2
KALRN		A_51_P215828	kalirin, RhoGEF kinase
KCNC1		A_52_P592305	potassium voltage gated channel, Shaw-related subfamily, member 1
KCNC2		A_51_P507333	potassium voltage gated channel, Shaw-related subfamily, member 2
KCNMA1		A_51_P187352	potassium large conductance calcium-activated channel, subfamily M, alpha member 1
KIAA0895L		A_51_P105241	KIAA0895-like
KIAA1549L		A_51_P519040	KIAA1549-like
KIAA1841		A_52_P434639	RIKEN cDNA 0610010F05 gene
KIF21B		A_52_P282500	kinesin family member 21B
KIF2A		A_52_P582519	kinesin family member 2A
KIF5C	x	A_52_P125429	kinesin family member 5C(Kif5c)
KIFAP3		A_52_P363301	kinesin-associated protein 3(Kifap3)
KIT		A_51_P355906	kit oncogene
LANCL2		A_52_P474665	LanC (bacterial lantibiotic synthetase component C)-like 2(Lancl2)
LIN7A		A_52_P48762	lin-7 homolog A (C. elegans)(Lin7a)
LRP8		A_51_P445765	low density lipoprotein receptor-related protein 8, apolipoprotein e receptor
LRRC17		A_51_P301930	leucine rich repeat containing 17(Lrrc17)
LRRC3B		A_51_P362089	leucine rich repeat containing 3B
MACROD2		A_51_P289464	predicted gene 2348; MACRO domain containing 2
MAP1B		A_51_P205139	microtubule-associated protein 1B(Map1b)
MAP2		A_51_P180754	microtubule-associated protein 2(Map2)
MAP2K6		A_52_P395242	mitogen-activated protein kinase kinase 6
MAP3K10		A_51_P377667	mitogen-activated protein kinase kinase kinase 10(Map3k10)
MAP3K9		A_52_P980158	mitogen-activated protein kinase kinase kinase 9(Map3k9)
MAP9		A_51_P250017	microtubule-associated protein 9
MAPK8		A_52_P558382	mitogen-activated protein kinase 8
MARK1		A_51_P295757	MAP/microtubule affinity-regulating kinase 1
MEF2C		A_51_P135802	myocyte enhancer factor 2C(Mef2c)
Meg3		A_52_P196568	maternally expressed 3
MET	x	A_52_P656418	met proto-oncogene(Met)

MGST3	A_51_P336850	microsomal glutathione S-transferase 3
Mlip	A_51_P427828	muscular LMNA-interacting protein
MMD	A_51_P431470	monocyte to macrophage differentiation-associated(Mmd)
MYCN	A_52_P536494	v-myc myelocytomatosis viral related oncogene, neuroblastoma derived (avian)
MYO1B	A_52_P289325	myosin IB
MYT1L	A_52_P499453	myelin transcription factor 1-like(Myt1l)
NCAM1	A_52_P37077	neural cell adhesion molecule 1
NEDD4L	A_52_P544878	neural precursor cell expressed, developmentally down-regulated gene 4-like
NEFL	A_51_P168646	neurofilament, light polypeptide(Nefl)
Nefm	A_51_P145220	neurofilament, medium polypeptide
NEGR1	A_52_P191412	neuronal growth regulator 1
NETO1	A_51_P145948	neuropilin (NRP) and tolloid (TLL)-like 1(Neto1)
NEUROD2	A_52_P624475	neurogenic differentiation 2
NLGN1	A_52_P497105	neuroligin 1(Nlgn1)
NOL4	A_52_P167317	nucleolar protein 4
Nos1ap	A_52_P491616	nitric oxide synthase 1 (neuronal) adaptor protein(Nos1ap)
NPAS4	A_51_P433810	neuronal PAS domain protein 4
NR4A1	A_51_P239654	nuclear receptor subfamily 4, group A, member 1
NREP	A_52_P301821	neuronal regeneration related protein(Nrep)
NRIP1	A_51_P152747	nuclear receptor interacting protein 1
NRXN1	A_52_P131476	neurexin I(Nrxn1)
Nrxn3	A_52_P39644	neurexin III
NTS	A_51_P485756	neurotensin(Nts)
PAK5	A_52_P629112	p21 protein (Cdc42/Rac)-activated kinase 3
PAK7	A_51_P107934	p21 protein (Cdc42/Rac)-activated kinase 7
PCLO	A_52_P141641	piccolo (presynaptic cytomatrix protein)(Pclo)
PDE10A	A_52_P972559	phosphodiesterase 10A(Pde10a)
PDE2A	A_51_P120024	phosphodiesterase 2A, cGMP-stimulated(Pde2a)
PGBD5	A_51_P343818	
PGM2L1	A_52_P301079	phosphoglucomutase 2-like 1
PIP4K2B	A_51_P267375	phosphatidylinositol-5-phosphate 4-kinase, type II, beta(Pip4k2b)
PLCB1	A_52_P506930	phospholipase C, beta 1
PLCXD2	A_51_P110529	plexin A2
PLXNA2	A_52_P431981	plexin A2(Plxna2)
PLXNA3	A_52_P539632	plexin A3(Plxna3)
PPM1E	A_51_P432024	protein phosphatase 1E (PP2C domain containing)(Ppm1e)
PPP3CB	A_52_P167125	protein phosphatase 3, catalytic subunit, beta isoform(Ppp3cb)
PRICKLE2	A_52_P29987	prickle-like 2 (Drosophila)
PRKCE	A_52_P375455	RIKEN cDNA 9630025F12 gene; protein kinase C, epsilon
PROK2	A_52_P158476	prokineticin 2(Prok2)
PROKR2	A_51_P396719	prokineticin receptor 2(Prokr2)
PTBP2	A_52_P593110	polypyrimidine tract binding protein 2(Ptbp2)
PTHLH	A_51_P129363	parathyroid hormone-like peptide(Pthlh)
PTPN5	A_51_P116239	protein tyrosine phosphatase, non-receptor type 5
PURG	A_52_P134317	purine-rich element binding protein G
PVRL3	A_52_P15495	poliovirus receptor-related 3
RAB3C	A_51_P481978	similar to RAB3C, member RAS oncogene family; RAB3C, member RAS oncogene family
RAB6B	A_51_P276418	RAB6B, member RAS oncogene family
RAB9B	A_52_P327993	RAB9B, member RAS oncogene family
RABGAP1L	A_51_P286610	RAB GTPase activating protein 1-like
RAP1GAP2	A_52_P39756	GTPase activating RANGAP domain-like 4
RAPGEF4	A_52_P731018	Rap guanine nucleotide exchange factor (GEF) 4(Rapgef4)
RBFOX2	A_52_P310530	RNA binding motif protein 9
RGS4	A_52_P43326	regulator of G-protein signaling4
ROBO1	A_51_P174314	roundabout homolog 1 (Drosophila)
RPRM	A_52_P627967	reprimo, TP53 dependent G2 arrest mediator candidate(Rprm)
RSPO2	A_51_P222337	R-spondin 2 homolog (Xenopus laevis)
RUFY3	A_52_P652328	RUN and FYVE domain containing 3(Rufy3)
RUNX1T1	A_51_P126206	runt-related transcription factor 1; translocated to, 1 (cyclin D-related)(Runx1t1)

RXRG		A_51_P513311	retinoid X receptor gamma
RYR2		A_52_P440709	ryanodine receptor 2, cardiac
SATB1		A_52_P110489	special AT-rich sequence binding protein 1(Satb1)
SCN3B		A_51_P184331	sodium channel, voltage-gated, type III, beta
SEMA3A		A_52_P527748	sema domain, immunoglobulin domain (Ig), short basic domain, secreted, (semaphorin) 3A(Sema3a)
SH3GL2		A_52_P1060059	SH3-domain GRB2-like 2(Sh3gl2)
SLA		A_52_P513177	src-like adaptor
SLC17A6		A_51_P174996	solute carrier family 17 (sodium-dependent inorganic phosphate cotransporter), member 6(Slc17a6)
SLC17A7		A_51_P137913	solute carrier family 17 (sodium-dependent inorganic phosphate cotransporter), member 7(Slc17a7)
Slc6a15		A_52_P220440	solute carrier family 6 (neurotransmitter transporter), member 15
SLC6A17		A_52_P373637	solute carrier family 6 (neurotransmitter transporter), member 17
SLC7A3		A_51_P275016	solute carrier family 7 (cationic amino acid transporter, y+ system), member 3
SLC8A1		A_51_P277373	solute carrier family 8 (sodium/calcium exchanger), member 1
SLF1		A_51_P207411	SMC5-SMC6 complex localization factor 1(Slf1)
SMARCA2		A_51_P504037	SWI/SNF related, matrix associated, actin dependent regulator of chromatin, subfamily a, member 2(Smarca2)
SNAP25		A_52_P165961	synaptosomal-associated protein 25
SNAPC3		A_51_P463270	small nuclear RNA activating complex, polypeptide 3
Snhg11		A_52_P70888	
SNRPN		A_52_P354390	small nuclear ribonucleoprotein N; SNRPN upstream reading frame; predicted gene 5802; similar to SNRPN upstream reading frame protein
SOCS2		A_52_P84037	suppressor of cytokine signaling 2; predicted gene 8000
SOX11		A_52_P160955	SRY (sex determining region Y)-box 11
SPOCK2		A_52_P393181	sparc/osteonectin, cwcv and kazal-like domains proteoglycan 2(Spock2)
SRGAP1		A_52_P477668	SLIT-ROBO Rho GTPase activating protein 1(Srgap1)
SSBP2		A_52_P557098	single-stranded DNA binding protein 2(Ssbp2)
ST8SIA4		A_51_P236267	ST8 alpha-N-acetyl-neuraminide alpha-2,8-sialyltransferase 4(St8sia4)
STAC3		A_51_P177261	SH3 and cysteine rich domain 3
STAU2		A_52_P24896	staufen (RNA binding protein) homolog 2 (Drosophila)
STC1		A_52_P189970	stanniocalcin 1
STMN2		A_51_P164998	stathmin-like 2
STRBP		A_52_P634051	spermatid perinuclear RNA binding protein
SV2A		A_52_P202142	synaptic vesicle glycoprotein 2 a
SYNPO	x		synaptopodin(Synpo)
SYP		A_51_P379428	synaptophysin
SYT1		A_52_P679799	synaptotagmin I(Syt1)
SYT16		A_51_P122867	synaptotagmin XVI(Syt16)
SYT4		A_52_P624302	synaptotagmin IV
TAC1		A_51_P216456	tachykinin 1
TANC2		A_52_P442986	tetratricopeptide repeat, ankyrin repeat and coiled-coil containing 2(Tanc2)
TBC1D16		A_51_P412225	TBC1 domain family, member 16(Tbc1d16)
TBC1D30		A_51_P262616	TBC1 domain family, member 30(Tbc1d30)
TBC1D9		A_52_P683580	TBC1 domain family, member 9
TBR1		A_51_P267017	T-box brain gene 1
TENM2		A_52_P472353	teneurin transmembrane protein 2(Tenm2)
TENM3		A_51_P383369	odd Oz/ten-m homolog 3 (Drosophila)
TENM4		A_52_P206429	teneurin transmembrane protein 4(Tenm4)
TMEM150C		A_51_P154379	transmembrane protein 150C
TMEM44		A_52_P419290	transmembrane protein 44
TMOD2		A_51_P234488	tropomodulin 2
TRO		A_52_P72831	trophinin(Tro)
TRPC4		A_52_P65273	transient receptor potential cation channel, subfamily C, member 4(Trpc4)
TSPYL4		A_52_P448357	TSPY-like 4(Tspyl4)
TSPYL5		A_52_P500002	testis-specific protein, Y-encoded-like 5(Tspyl5)
UBE2D1		A_52_P546459	ubiquitin-conjugating enzyme E2D 1(Ube2d1)
UBQLN2		A_52_P381929	ubiquilin 2
VSNL1		A_52_P515880	visinin-like 1

VSTM2A	A_52_P365781	V-set and transmembrane domain containing 2A
WDR47	A_52_P124195	WD repeat domain 47
WIF1	A_51_P484526	Wnt inhibitory factor 1(Wif1)
ZDBF2	A_52_P571561	zinc finger, DBF-type containing 2
ZNF385B	A_51_P451275	similar to zinc finger protein 533; zinc finger protein 385B
ZNF711	A_52_P753819	zinc finger protein 711
ZNF846	A_52_P589664	
